# Supplementary material for: Molecular mechanisms and topological consequences of drastic chromosomal rearrangements of muntjac deer
Source: Nat Commun. 2021 Nov 25;12:6858. doi: 10.1038/s41467-021-27091-0 (PMC8617201; doi:10.1038/s41467-021-27091-0)
Supplement: Supplementary file 1 — Supplementary Information [file 41467_2021_27091_MOESM1_ESM.pdf]

Supplementary Information for

Molecular mechanisms and topological consequences of drastic chromosomal rearrangements of muntjac deer

Yuan Yin#, Huizhong Fan#, Botong Zhou#, Yibo Hu#, Guangyi Fan#, Jinhuan Wang#, Fan Zhou#, Wenhui Nie#, Chenzhou Zhang, Lin Liu, Zhenyu Zhong, Wenbo Zhu, Guichun Liu, Zeshan Lin, Chang Liu, Jiong Zhou, Guangping Huang, Zihe Li, Jianping Yu, Yaolei Zhang, Yue Yang, Bingzhao Zhuo, Baowei Zhang, Jiang Chang, Haiyuan Qian, Yingmei Peng, Xianqing Chen, Lei Chen, Zhipeng Li, Qi Zhou*, Wen Wang*, Fuwen Wei*

#These authors contributed equally to this work.

*Corresponding author. E-mail: weifw@ioz.ac.cn (F.W.); wenwang@nwpu.edu.cn (W.W.); zhouqi1982@zju.edu.cn (Q.Z.);

This Supplementary Information includes:

Supplementary Notes

Supplementary Figures 1-27

Supplementary Tables 1-18

Supplementary Notes:

Samples collection

In this study, we collected various types of samples from five species, including *Hydropotes inermis*, *Muntiacus reevesi*, *M. gongshanensis*, *M. crinifrons*, and *Elaphurus davidianus*. The blood samples of two female *M. crinifrons* individuals (female MCR1, female MCR2), two male *M. crinifrons* individuals (male MCR1,male MCR2) and one male *M. reevesi* individual (MRE1) were collected from Hefei Wild-life Zoo, Hefei City, Anhui province, China. The blood sample of one *H. inermis* individual (HIN1) were collected from a farm in Yancheng, Jiangsu province, China. The blood sample of one *E. davidianus* was collected from the Beijing Milu Ecological Research Center. In addition, the frozen tissue samples of a female *M. crinifrons* individual (female MCR3), a male *M. crinifrons* individual (male MCR3), two *M. gongshanensis* individuals (MGO1, MGO2), the fibroblast cell lines of the skin sample from a male *M. crinifrons* individual (male MCR4) and the lung sample from a female *M. crinifrons* individual (female MCR4) were provided by Kunming Cell Bank of the Chinese Academy of Sciences (**Supplementary Table 1**).

All animal specimens were collected legally in accordance to the policy of Animal Care and Use ethics of each institution, which meets or exceeds US regulatory standards for the humane care and treatment of animals in research.

Library construction and Sequencing

The high-quality DNA of above samples was extracted using a DNeasy Blood & Tissue Kit (Qiagen, Valencia, CA, USA) Qiagen Kit according to the manufacturer's instructions. Then these DNA samples were used to construct different libraries for Nanopore, NGS and Hi-C sequencing. The Nanopore libraries with an insert size of 20 kb were sequenced using R9.4 flow cells on the GridION X5 sequencer (ONT, UK) at the Genome Center of Grandomics (Wuhan, China). The ONT Albacore software was used to perform the base calling, adaptor removal and low-quality base filtration on

fast5 files. The NGS libraries with an insert size of 400bp was constructed and then sequenced on Illumina NovaSeq platform with the paired-end read length of 150bp. The Hi-C libraries were constructed and sequenced using Illumina NovaSeq platform. The information for clean data of Nanopore, NGS and Hi-C sequencing are listed in Supplementary Table 2-**4**, respectively.

mRNAs were isolated from the two cell line samples of *M. crinifrons* according to the TRIzol (Invitrogen, USA) protocol. Sequencing libraries for 150 bp paired-end reads were then generated and sequenced using an Illumina HiSeq platform. Finally, a total of 6 Gb paired-end RNA-seq sequencing data were generated for each sample.

Assembly of contig-level genome

In this study, we firstly assembled the contig-level genomes for *M. reevesi*, *M. gongshanensis*, female and male *M. crinifrons* using long reads and then polished them using high-quality Illumina short reads. Briefly, for the genomes of *M. reevesi* and female *M. crinifrons*, the nanopore long reads from individual MRE1 and female MCR1 were firstly assembled using SMARTdenovo [1] (veriosn 1.0, with parameter -k 21 -J 3000 -t 20), and then the paired-end Illumina reads from the same individuals were used to polish the genomes using Nextpolish software [2] (version1.2.4, with parameter best). For the genome of *M. gongshanensis*, the nanopore long reads of individual MGO1 were firstly assembled using the SMARTdenovo [1], then the Racon software [3] (version 1.21 with default parameter) and Pilon software [4] (version 1.23 with default parameter) were used to polish the genome using raw nanopore reads and high-quality Illumina reads. For the genome of male *M. crinifrons*, the PacBio reads were downloaded from NCBI (PRJNA438286) and reassembled using wtdbg2 [5]

(version 2.5, with default parameter), then the Racon and Pilon software [4] were used to polish this assembly using long reads and Illumina reads, respectively. The statistics of these draft assemblies are listed in **Supplementary Table 5**. The genome size of these muntjac species are similar to that assembled using Illumina reads by Chen et al. [6].

Chromosome assembly by Hi-C data

We improved the newly assembled genomes of *M. reevesi*, female and male *M. crinifrons* and previous published *H. inermis* genome downloaded from NCBI (GCA_006459105.1) to chromosome level using 52~71 folds high-quality high throughput chromatin conformation capture (Hi-C) data (**Supplementary Table 4**). Firstly, the paired-end Hi-C reads of female and male *M. crinifrons*, *M. reevesi* and *H. inermis* were aligned to their contig-level draft genomes using Juicer software [7] (version 1.5.7). Then the 3D-DNA software [8] (version 180922) was used to order and orientate the contigs. Finally, the Juicebox Assembly Tools [9] was used to help manually correct the position and orientation of contigs based on the Hi-C contacted heatmaps. The statistics of these chromosome-level genomes were listed in **Supplementary Table 5**. The chromosome 1p+4 (neo-Y chromosome) of male *M. crinifrons* were manually adjusted using male-specific mutations.

Synteny with *B. taurus* genome

We performed the genome synteny analysis by aligning the five genomes we assembled to *B. taurus* genome (ARS-UCD1.2) using LAST software [10] (version 885). Briefly, each genome was firstly aligned to *B. taurus* genome using "lastal" command with parameter: -E0.05. Then the "maf-swap" command was used to change the order of the sequences in MAF-format and the best pairwise aligned blocks was obtained by using “last-split” command with the parameter: -m1. The synteny relationships between the these genomes and *B. taurus* genome are presented

by Circos (version 0.69-6) [11] after filtering < 2 kb alignment segments.

Repeat Annotation

To predict gene models, various type of repeats should be firstly identified and masked. Tandem repeats were annotated by Tandem Repeat Finder (TRF) [12] (version 4.07b; with parameters: 2 7 7 80 10 50 2000 -d -h). Long terminal repeat retrotransposons (LTR elements) were found out by LTR_FINDER (version 1.0.5) [13]. The RepeatModeler [14] (version 1.0.4) software was used to build *de novo* repeat library. Transposable elements (TE) on genomes were annotated by mapping TE from the *de novo* repeat library and the Repbase TE library [15] (version 16.02) against genomes using RepeatMasker [14] (version 4.0.5; with parameters: -nolow -no_is -norna -parallel 1). TE-relevant proteins were identified by RepeatProteinMask software (a package in RepeatMasker) (version open-4.0.6, with parameters: -noLowSimple -p value 0.0001).

Gene annotation

We used the combined pipeline of *de novo* and homology-based method to predict genes as the Ruminant Project [6]. In brief, SNAP (version 2006-07-28) [16], GENSCAN (version 1.0) [17], GlimmerHMM (version 3.0.4) [18], and AUGUSTUS (version 2.5.5; Augustus: Gene Prediction) [19] were separately used to annotated gene models *de novo* based on the repeat-masked genomes. For homology-based predictions, the protein sequences of *Homo sapiens*[20] (Ensemble 87 release), *B. taurus* [21] (Ensemble 87 release) and *Ovis aries* [21] (Ensemble 87 release) were used as templates. First, the protein sequences were aligned to the genomes by TBLASTN [22] with an E-value cut-off of 1e-5. The blast hits were transformed into candidate gene locus with GenBlastA [23]. Then the genomic sequences of candidate gene loci were extracted and subjected to perform more precise alignment using GeneWise (version 2.2.0) [24]. The EVidenceModeler software [25] (EVM, version 1.1.1) was used to integrate the gene

models predicted by *de novo* and homology approaches. Completeness evaluation of gene annotation was carried out using BUSCO [26] (version 3.0.2). The function of protein coding genes were annotated by aligning their protein sequence to the SwissProt and KEGG database using BLAST [27] (version 2.2.26; parameter: blastall -p blastp -b 100 -v 100 -e 1e-5 -m 8). When a gene is aligned by multiple proteins in SwissProt or KEGG database, the one with the highest blast score is remained.

Delineating the regions of fusion sites

For each chromosome of female *M. crinifrons*, we used an in-house python script to detect the regions of fusion site which were defined as the interval regions between two adjacent large-scale syntenic blocks that aligned by different chromosomes of *B. taurus* or *H. inermis*. In this study, when the regions of the same fusion site inferred by *B. taurus* and *H. inermis* were different, the smaller one was selected in the subsequent analysis.

We used the following steps to counts the number of gaps in the fusion sites and in the whole genome, Firstly, the gap was set to be 500 Ns when the 3D-DNA [8] was used for chromosome assembly, so the number of gaps could be directly counted in whole genome and in the fusion site regions. A total of 261 gaps were identified in whole genome of female *M. crinifrons*, 35 of which are located in fusion site regions and their flanking 200 kb regions (200 kb upstream and downstream of each fusion site). Then, we mapped the Illumina reads of the individual female MCR2 to the reference genome using BWA [28] with default parameter and calculated the coverage of each locus using SAMtools (samtools depth -a) [29]. The region is regarded as a gap when the coverage of 100 consecutive loci are all zero. We found two more gaps using this strategy. Finally, we scanned the alignments of nanopore reads on the genome and found a connection error in the fusion site region chr2: 470,398,810-470,568,565, which was also regarded as a gap. In total, we found 264 gaps in the whole genome, 38 of which are located in the fusion site (**Supplementary Table 13**).

These results show that the average density of gaps is 2.0 per Mb and 0.09 per Mb in fusion site region and the rest genomic regions, respectively. For the 31 fusion site regions, only one (chr2: 61,703,828-62,110,032) does not contain any gap which could be confirmed by the accurate alignment of nanopore reads at this fusion site **supplementary Fig. 19** [30].

Extracting monomers of three satellite sequences specific to Cervidae from female *M. crinifrons* genome

To calculate the content of satellite and telomeric sequences in the fusion site regions, we firstly downloaded three classes of Cervidae-specific satellite sequence cloned by Liu et al. in *M. crinifrons* [31]. Then by exploring the homologous sequences of these downloaded satellite sequence in the tandem repeat annotation results of our female *M. crinifrons* genome, two types monomers for satellite I (satI_980 and satI_790) and monomer for satellite II (satII) and satellite IV (satIV) were identified.

Supplementary Figures

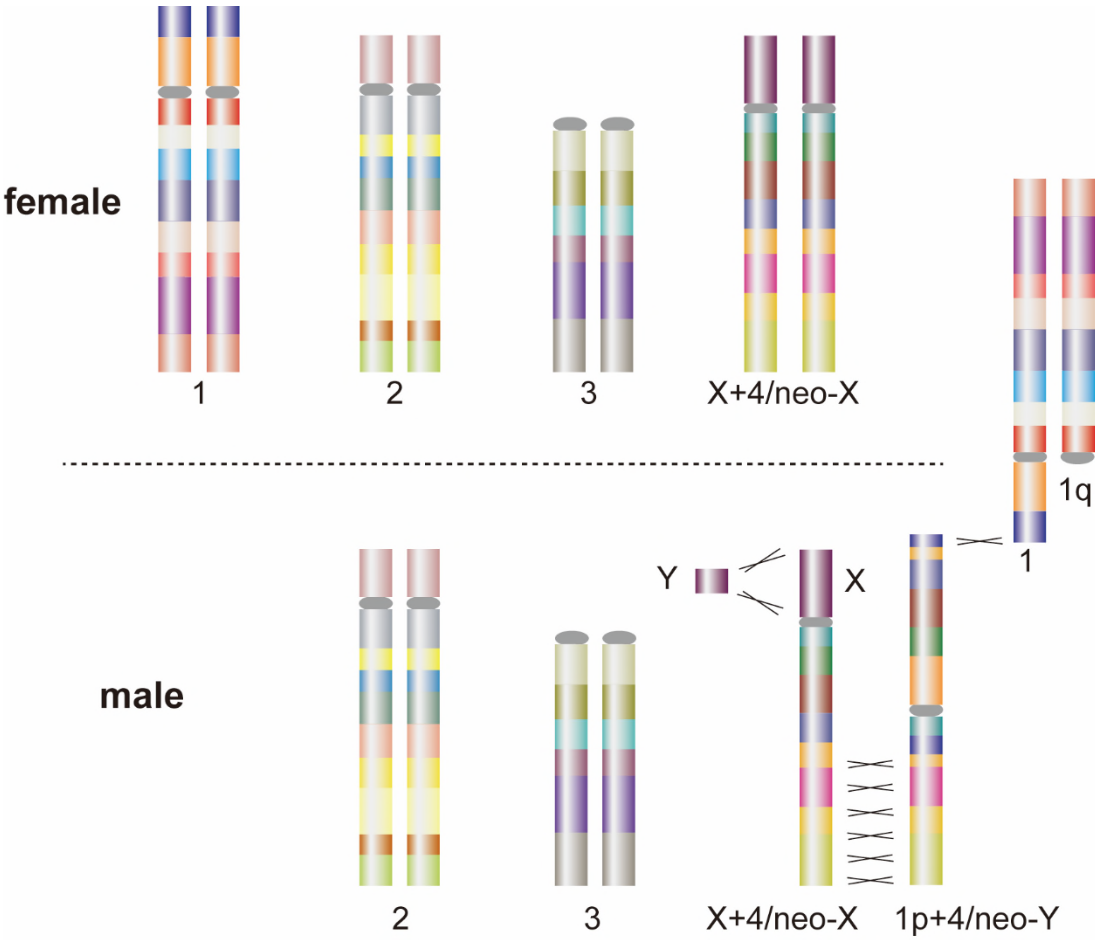

Supplementary Figure 1. Karyotype pairing diagram of *M. crinifrons*. Segments with different colors represent different ancestral chromosomes. Gray ellipses represent centromeres. The cross between chromosome Y, X+4, 1p+4, 1q in male *M. crinifrons* indicates their pairing relationship. 1p and 1q mean short arm and long arm of chromosome 1, respectively.

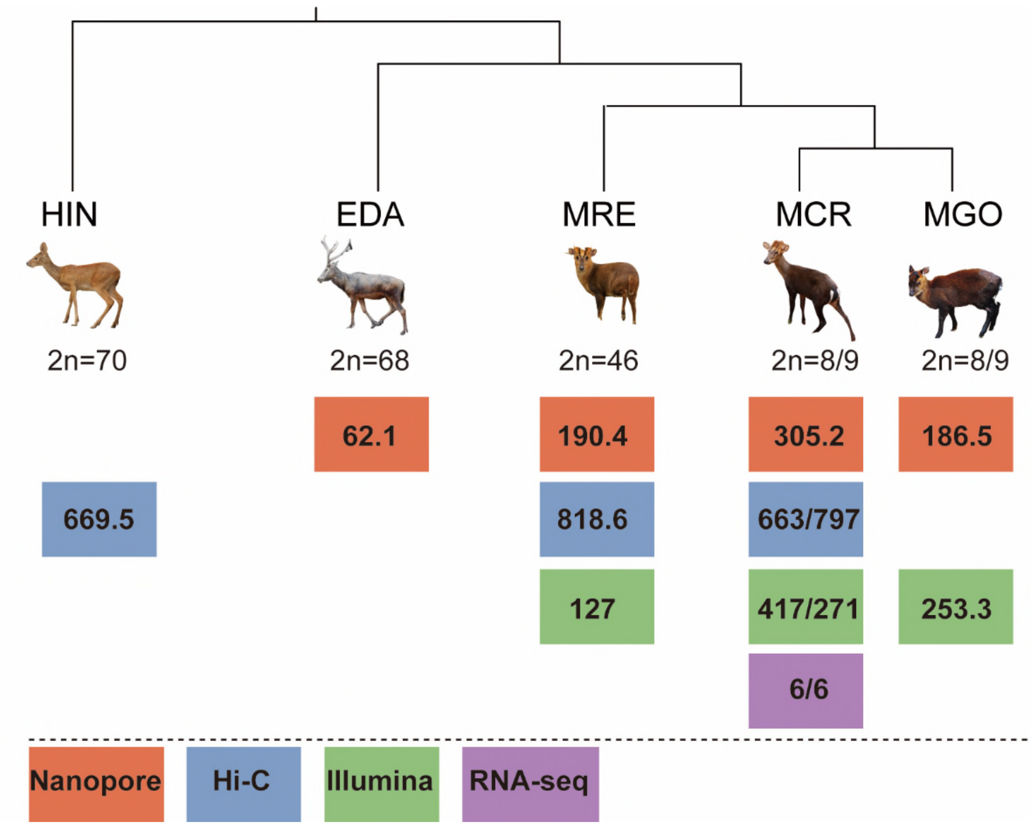

Supplementary Figure 2. Summarized information of species, sequencing technology and amount of data in this study. The amount of data is in Gb. HIN, *H. inermis*; EDA, *E. davidianus*; MRE, *M. reevesi*; MGO, *M. gongshanensis*; MCR, *M. crinifrons*. The data of female and male *M. crinifrons* are counted separately and showed by diagonal lines. Detailed information can be found in the Supplementary Table 1-4.

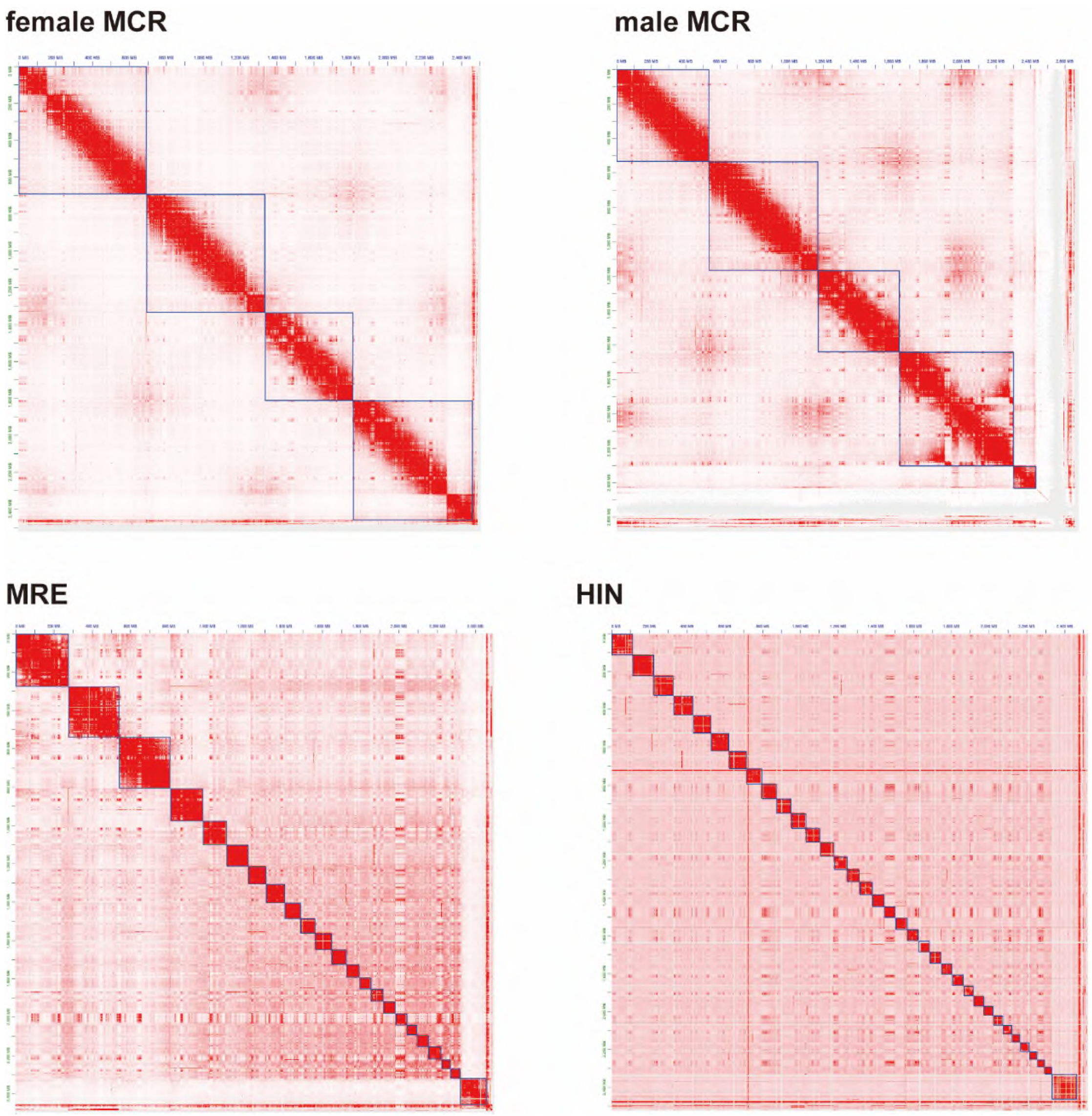

Supplementary Figure 3. Heatmap of Hi-C interactions on chromosomes-level genomes. Each chromosome is framed in blue box. From top left to bottom right are the chromosome 1, 2, 3 and X+4 of female *M. crinifrons* (female MCR), the chromosome 1q, 2, 3, 1p+4 and X of male *M. crinifrons* (male MCR), the chromosome 1-22 and X of *M. reevesi* (MRE) and the chromosome 1-34 and X of *H. inermis* (HIN), respectively. 1p and 1q represent short arm and long arm of chromosome 1, respectively.

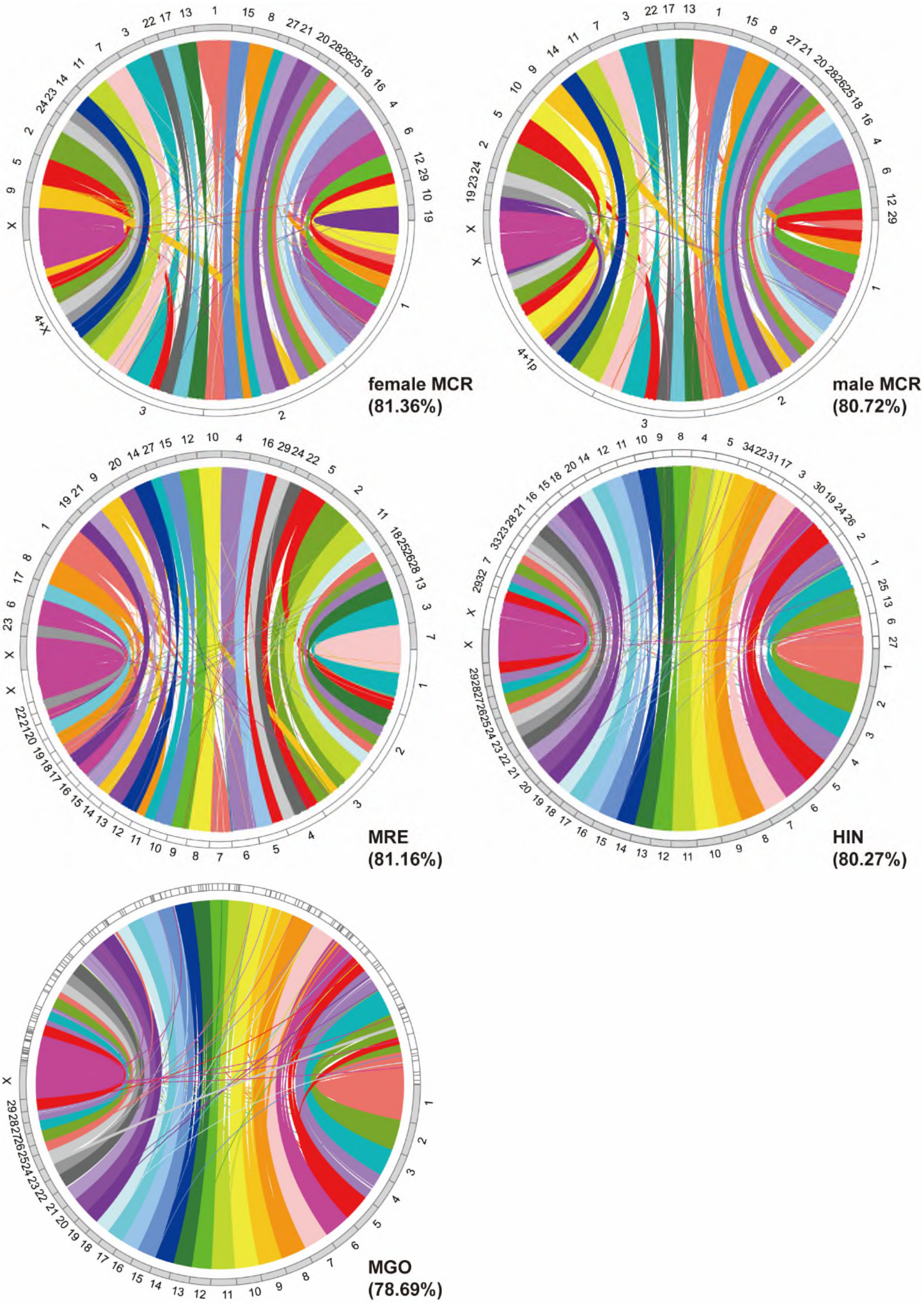

Supplementary Figure 4. Circos plot showing alignments of five genomes we assembled against to *B. taurus* genome. The alignments less than 2kb were discarded for clearer displaying. Gray blocks represent *B. taurus*'s chromosomes, while white blocks represent chromosomes of female and male *M. crinifrons* (MCR),

M. reevesi (MRE) and *H. inermis* (HIN) or contigs of *M. gongshanensis* (MGO). Numbers near chromosome blocks represent chromosome codes. The percentage in brackets indicates proportion of sequence in *B. taurus* genome aligned by each genome.

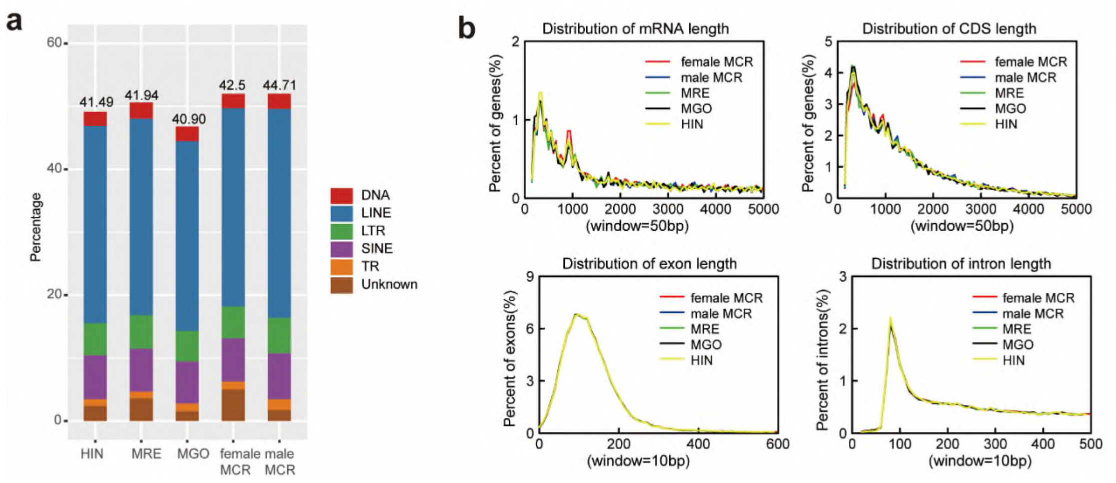

Supplementary Figure 5. Genome annotation. a Content of annotated repetitive sequence. The percentage of total repetitive sequence in each genome is showed above the pillar. The total percentage value is not equal to the sum of percentage of different types repeats due to the overlap of them. HIN, *H. inermis*; MRE, *M. reevesi*; MGO, *M. gongshanensis*; MCR, *M. crinifrons*. **b** Statistical results of annotated protein coding genes.

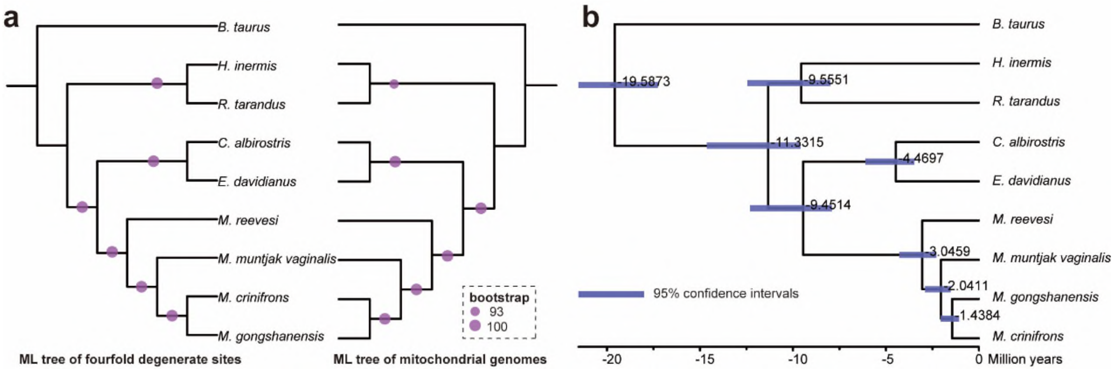

Supplementary Figure 6. Phylogeny and divergent time. a Consistent maximum likelihood (ML) phylogenetic tree from fourfold degenerate sites (4dTV) and mitochondrial genomes. 200 and 1000 bootstrap replicates were used to calculate the support for each node on 4dTV and mitochondrial genomes tree, respectively. The size of purple dots represents the percentage of bootstrap repeats supporting a node. **b** Divergence time of muntjac deer and outgroup species.

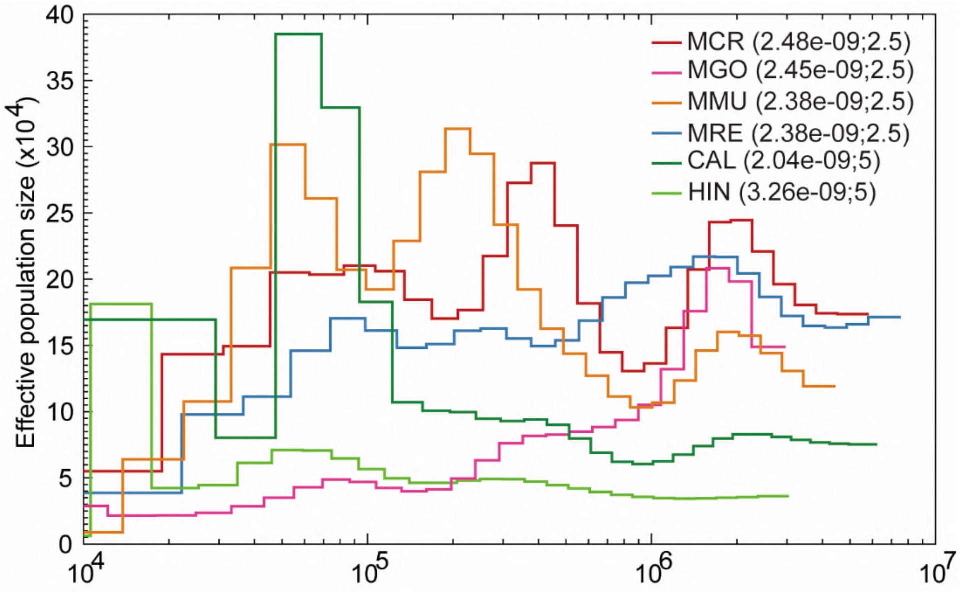

Supplementary Figure 7. Demographic history of muntjac deer and related Cervidae species. Mutation rate estimated by r8s and generation time are putted in parentheses after species id. MCR, *M. crinifrons*; MGO, *M. gongshanensis*; MMU, *M. muntjak vaginalis*; MRE, *M. reevesi*; CAL, *C. albirostris*; HIN, *H. inermis*.

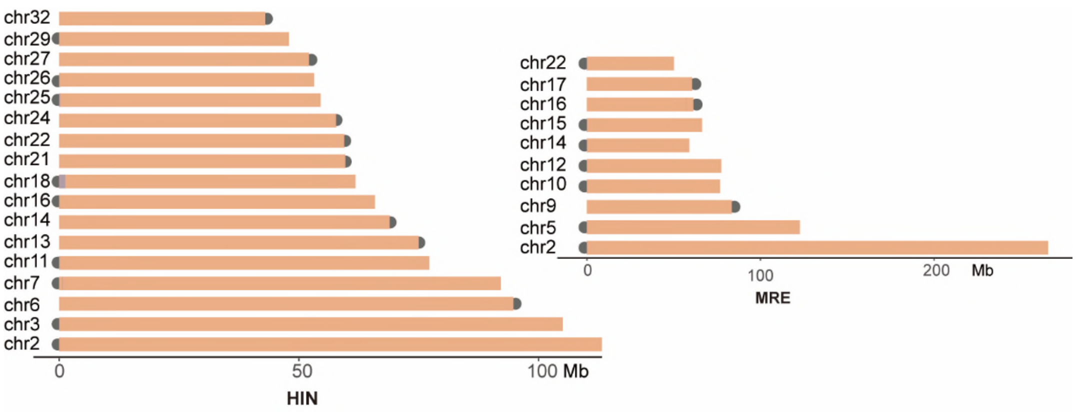

Supplementary Figure 8. Centromeric positions on some chromosomes of *H. inermis* (HIN) and *M. reevesi* (MRE). Gray semicircle represents centromere identified by scanning Cervidae-specific centromeric satellite sequences. Horizontal orange bars represent chromosomes with the chromosome code on the left.

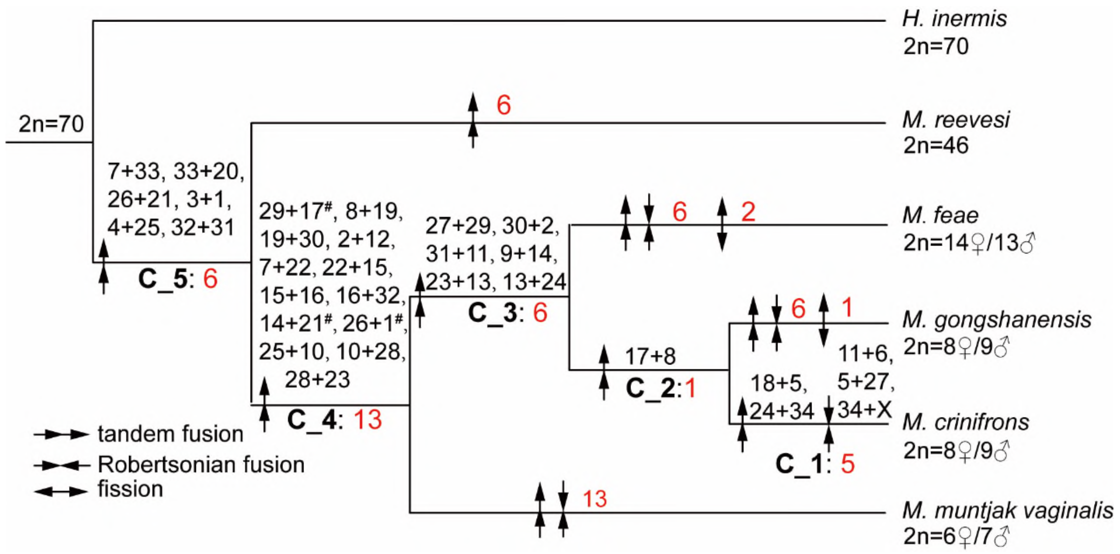

Supplementary Figure 9. Chromosome fusion events leading to the female *M. crinifrons*. The “+” indicates chromosome fusion events happened between its flanked ancestral chromosomes represented by chromosome names of *H. inermis*. The red numbers indicate the number of fusion or fission events. C_1~C_5 marked five chromosomal evolution stages in female *M. crinifrons*. Three fusion events in C_4 were marked using “#” because of their further fission in *M. feae* or *M. gongshanensis*.

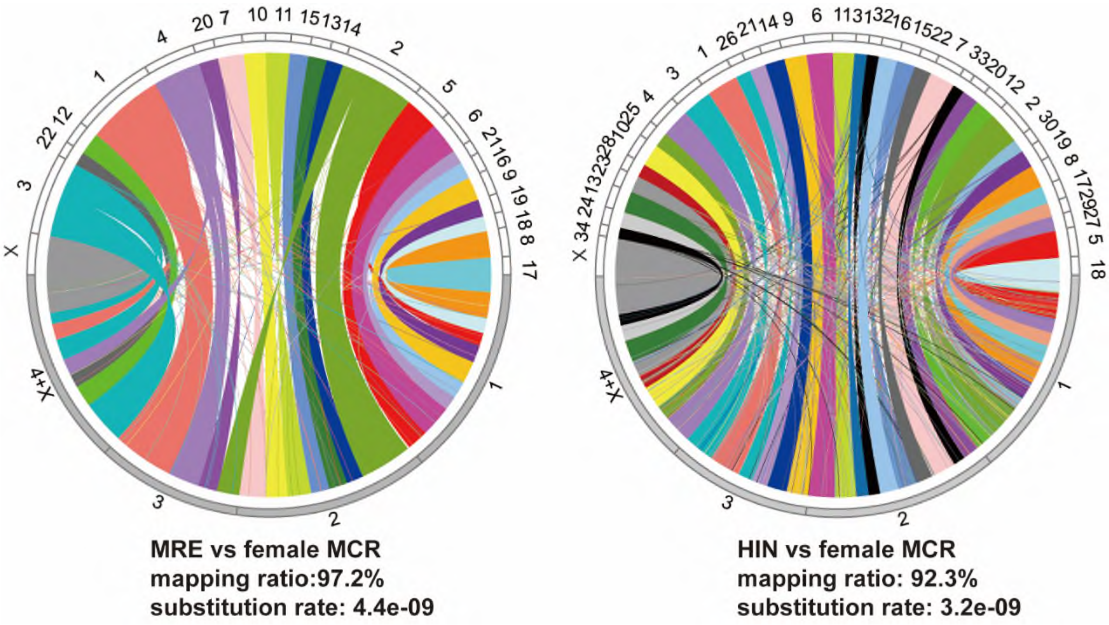

Supplementary Figure 10. Circos plot showing genome alignments of female *M. crinifrons* with its related species. Gray boxes indicate chromosomes of female *M. crinifrons* (MCR), and blank boxes indicate chromosomes of *H. inermis* (HIN) or *M. reevesi* (MRE). Numbers near chromosome blocks represent chromosome numbers.

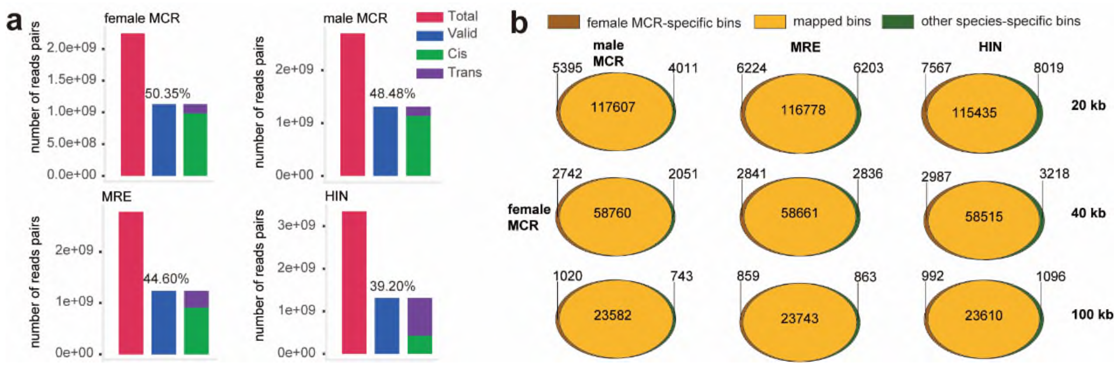

Supplementary Figure 11. Hi-C reads pairs and homologous bins across genomes. a Number of Hi-C reads pairs of female and male *M. crinifrons* (MCR), *M. reevesi* (MRE) and *H. inermis* (HIN). “Total” represents the total number of sequenced Hi-C reads pairs. “Valid” represents the remained valid reads pairs to construct contact matrix. Percentage of valid reads pairs were putted on the corresponding bars. “Cis” and “Trans” indicates valid reads pairs with their two reads aligned to the same chromosome and different chromosomes, respectively. **b** Number of mapped homologous bins between female *M. crinifrons* and other three genomes at 20 kb, 40 kb and 100 kb resolutions.

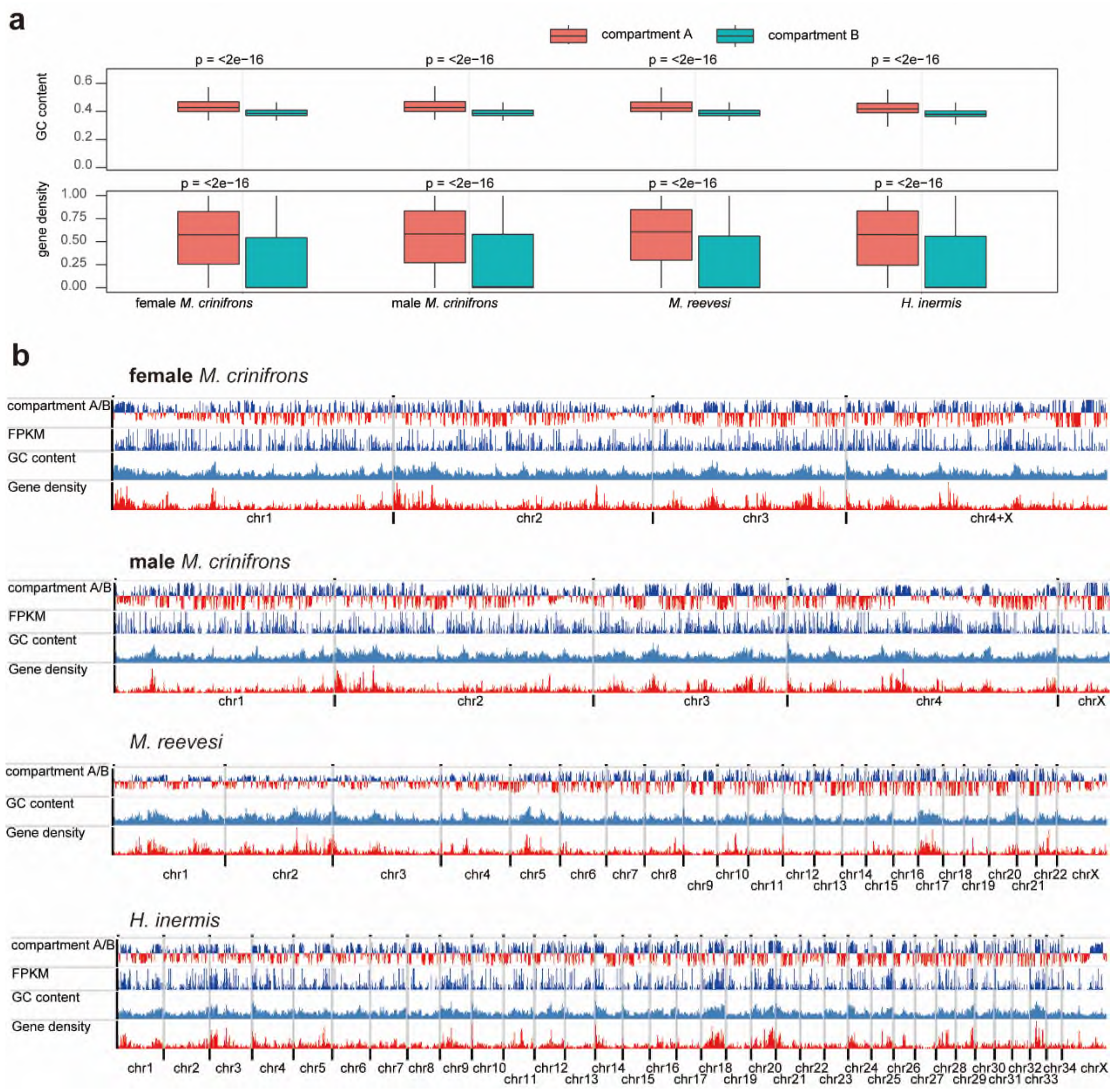

Supplementary Figure 12. Compartment A/B and corresponding genome sequence features. **a** Boxplot of gene density and GC content in genomic compartment A and B. In female and male *M. crinifrons*, *M. reevesi* and *H. inermis*, the number of bins belonging to compartment A and compartment B are respectively 11,600 and 12,510, 11,727 and 12,112, 12,266 and 11,848, 12,290 and 11,922. The lower and upper hinges correspond to the first and third quartiles. The horizontal line inside the box is the median. The upper whisker extends from the hinge to the largest value no further than 1.5 * IQR from the hinge (where IQR is distance between the first and third quartiles). The lower whisker extends from the hinge to the smallest value at most 1.5 * IQR of the hinge. Outlier are not displayed. The difference of gene density or GC content in different compartment regions was checked using

independent two-sample *t*-test (two sides and *p*-value <0.05) without multiple comparisons adjustments. **b** Compartment A/B, gene expression level, GC content and gene density of female *M. crinifrons*, male *M. crinifrons*, *M. reevesi* and *Hydropotes inermis*. In the track of compartment A/B, the blue prat represent the compartment A, and the red part represent compartment B. The gene expression level is represented by the FPKM (fragments per kilobase of transcript per million). GC content show the percentage of GC bases per 100 kb. Gene density is the total length ratio of genes per 100 kb. For male *M. crinifrons*, 1p and 1q represent short arm and long arm of chromosome 1, respectively.

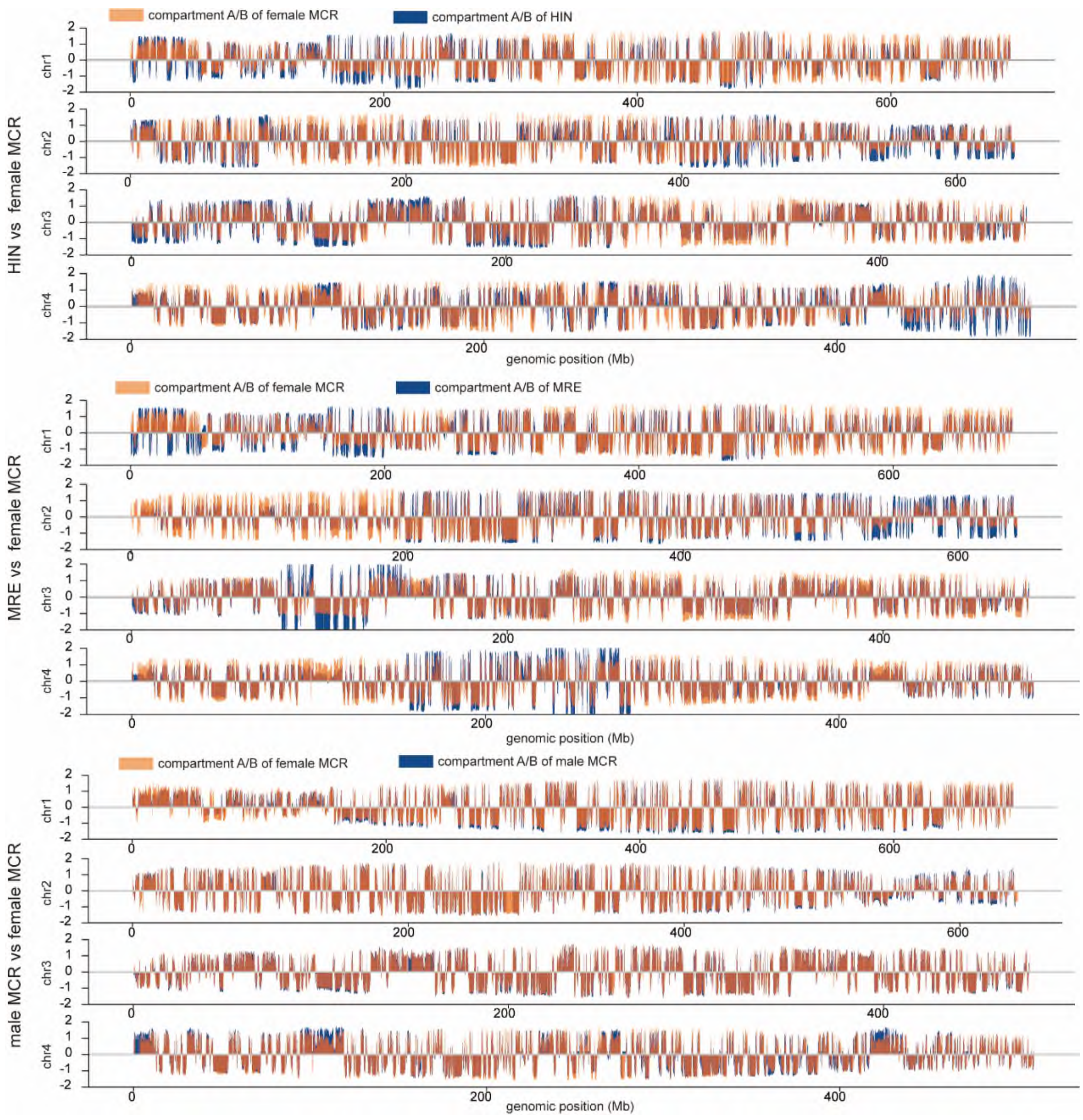

Supplementary Figure 13. Comparison of compartment A/B between female *M. crinifrons* and other three genomes. Compartment results of the three genomes were mapped to the genome of female *M. crinifrons* according their bins' homologous relationship. On the ordinate, the value greater than zero means compartment A, and that less than zero means compartment B. The transparent orange pillars represent the compartment results of female *M. crinifrons* and the dark blue ones represent that of other three genomes. MCR, *M. crinifrons*; MRE, *M. reevesi*; HIN, *H. inermis*.

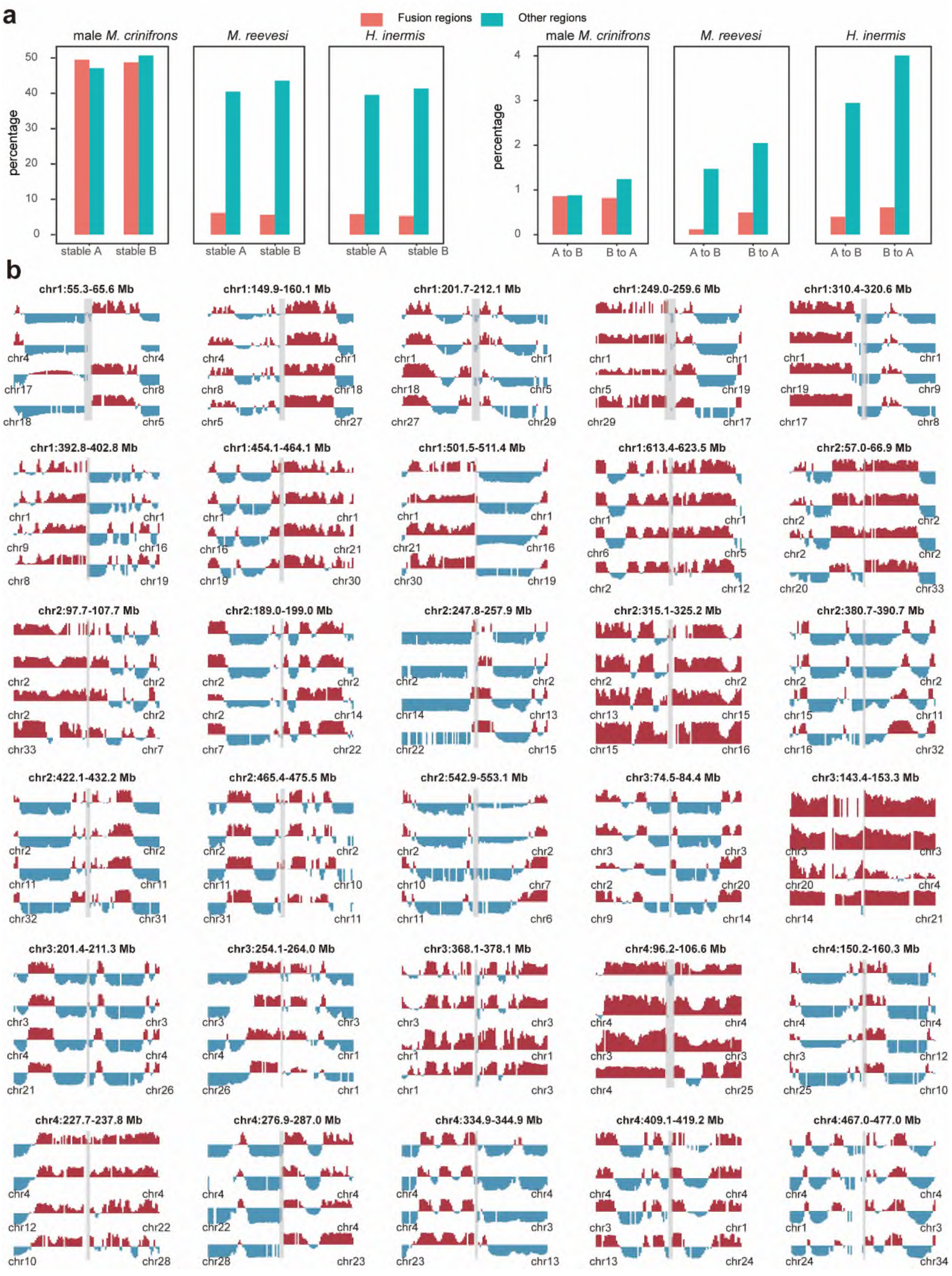

Supplementary Figure 14. Comparison of compartment A/B near fusion sites. a Percentage of bins with switched or stable compartment A/B between female and female *M. crinifrons*, *M. reevesi* and *H. inermis*. "Fusion regions" refers to female *M. crinifrons* fusion site regions and their upstream and downstream 5 Mb regions. "other regions" refers to the rest genomic regions except for the ancestral X chromosome

region. **b** Detailed compartment A/B in regions near fusion site of female *M. crinifrons* and their homologous regions in other three genomes. From top to bottom in each figure are female and male *M. crinifrons*, *M. reevesi* and *H. inermis*. Red and blue blocks indicates compartment A and compartment B, respectively. The gray transparent boxes mark the fusion sites. Under each graph of male *M. crinifrons, M. reevesi* and *H. inermis* are the chromosome names where these regions are located in their genomes.

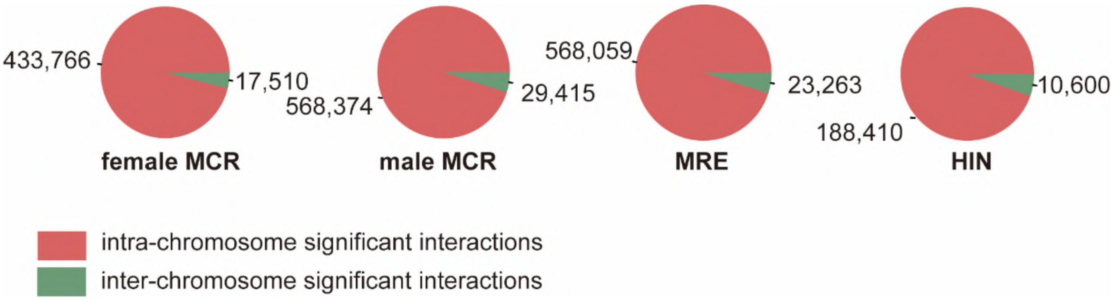

Supplementary Figure 15. Total significant interactions. Number and proportion of intra-chromosome and inter-chromosome significant interactions identified in female and male *M. crinifrons* (MCR), *M. reevesi* (MRE) and *H. inermis* (HIN).

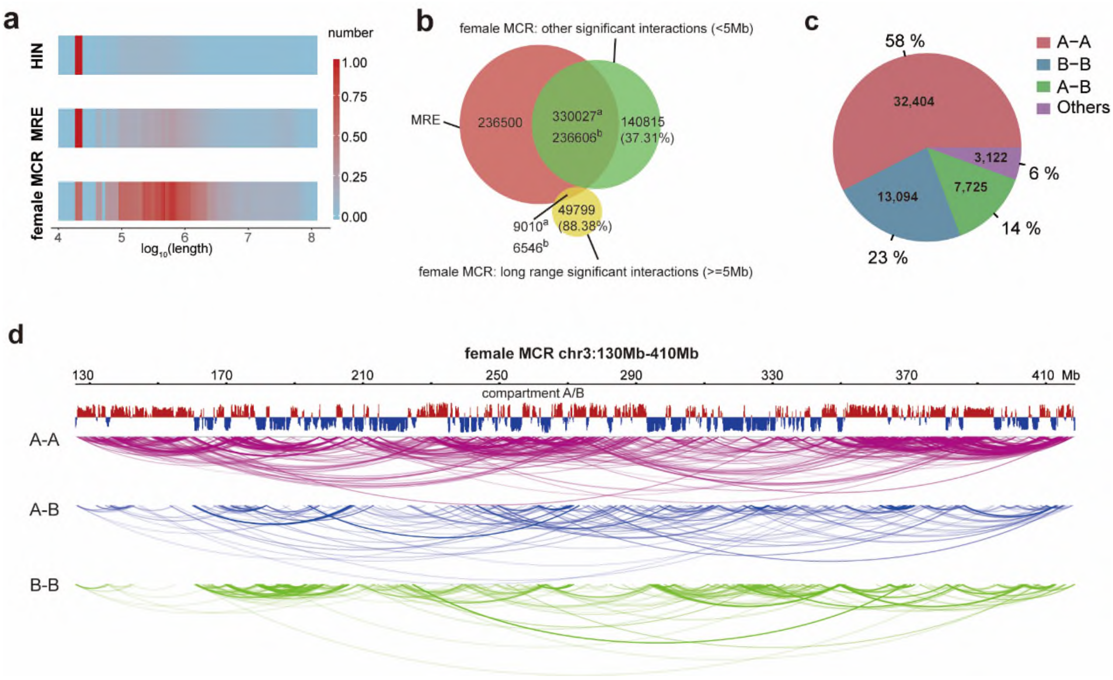

Supplementary Figure 16. Lang-range significant interactions. a Length distribution of intra-chromosome significant interactions. The length of significant interactions was transformed by $\log_{10}$ function. The number of significant interactions with different length in each genome was normalized by the maximum and minimum values, respectively. **b** Number of shared and specific significant interactions between *M. reevesi* and female *M. crinifrons*. Significant interactions of female *M. crinifrons* with length more than 5 Mb and less than 5 Mb were separately displayed. Superscript “a” indicates number of significant interactions of *M. reevesi* shared by female *M. crinifrons* and superscript “b” indicates number of significant interactions of female *M. crinifrons* shared by *M. reevesi*. In parentheses are the percentage of significant interaction specific to female *M. crinifrons*. **c** Number and percentage of long-range significant interactions (>= 5Mb) with various compartment type at their both ends in female *M. crinifrons*. “A-A”, long-range significant interactions (LRSI) with compartment A at their both ends; “B-B”, LRSI with compartment B at their both ends; “A-B”, LRSI with compartment A at one end and compartment B at another end; “Others”, LRSI with no exact compartment type at any of their two ends. **d** Compartment A/B and different type of LRSI in the region of chr3:130-410Mb in female *M. crinifrons*. The red bars mark compartment A and the blue ones mark

compartment B.

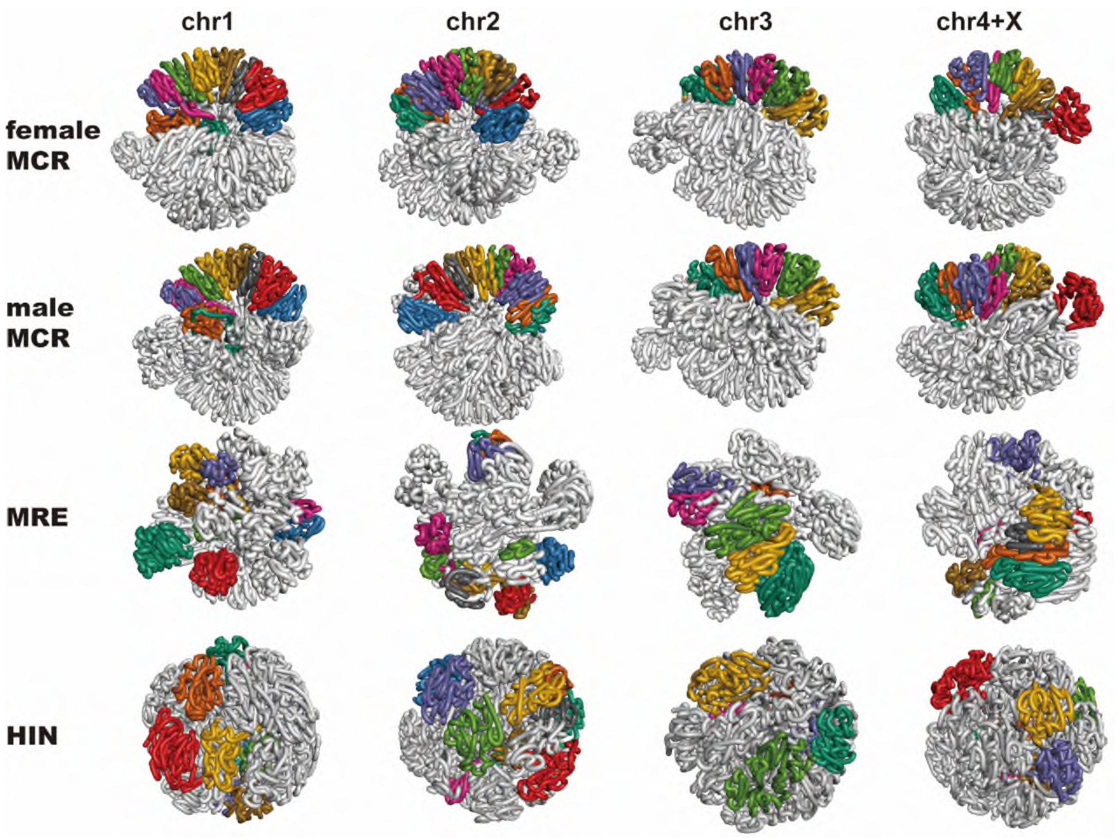

Supplementary Figure 17. 3D genome structure of different genomes. The “chr1”, “chr2”, “chr3” or “chr4+X” at top of each column are the chromosome codes of female *M. crinifrons*. In each column, only the corresponding chromosome of female *M. crinifrons* and its homologous chromosomes or segments in other genomes are colored, and the remaining chromosomes are gray. Segments of different colors in each column represent different ancestral chromosomes, while the same color indicates homologous ancestral chromosome segments in different genomes.

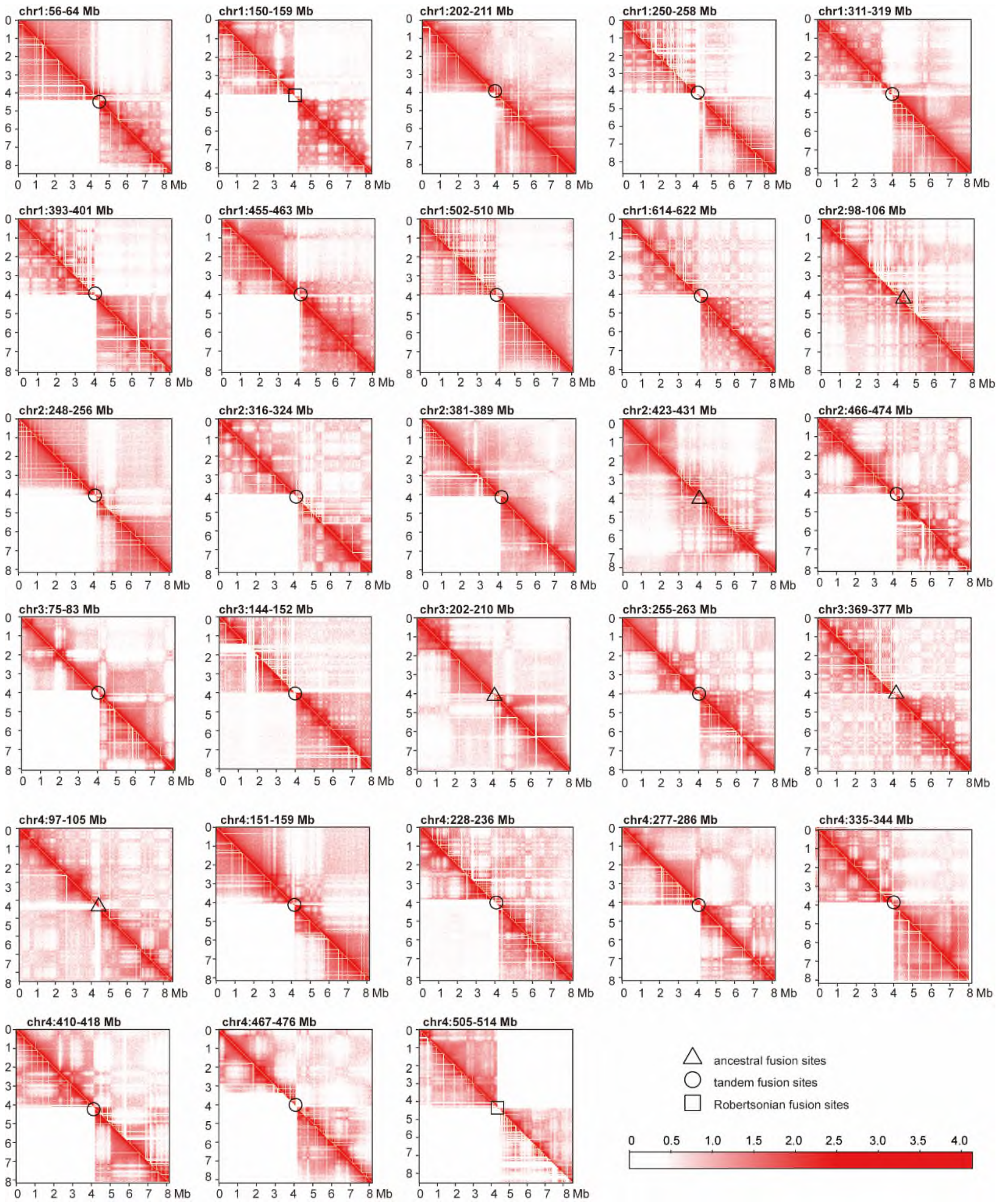

Supplementary Figure 18. Combined heatmaps of contact matrix around the fusion sites of female *M. crinifrons* (upper right) and their homologous regions in *M. reevesi* (lower left) at 20 kb resolution. Hollow geometries marks the location of fusion site. Different geometries represent different fusion site types. The "ancestral fusion sites" refers to the oldest fusion sites shared by five muntjac species. The "tandem fusion sites" represent the tandem fusion sites of female *M. crinifrons* except the ancestral fusion sites. The "Robertsonian fusion sites" refers to fusion sites raised by Robertsonian fusion and they are the youngest fusion sites.

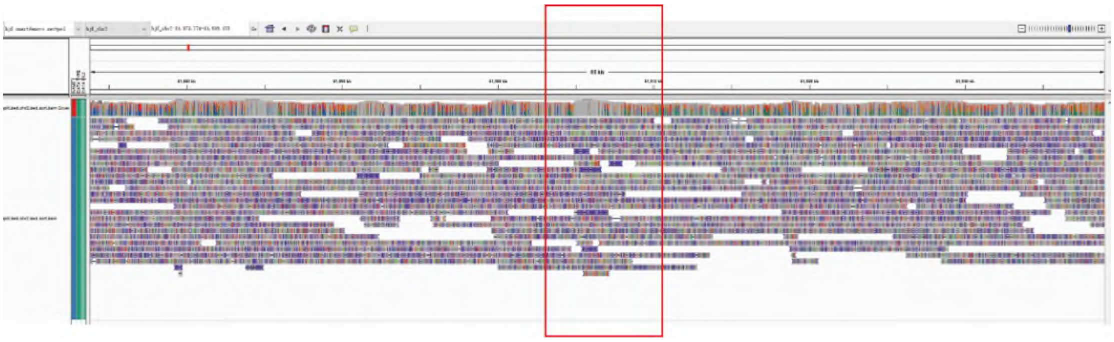

Supplementary Figure 19. Nanopore reads alignment results at the completely assembled fusion site. The alignment results of nanopore reads at the fusion site (chr2: 61,703,828-62,110,032) and its flanking regions are extracted from the Integrative Genomics Viewer (IGV). The red box demarcates the fusion site and its upstream and downstream 200 kb interval. Some reads can span the whole fusion site, suggesting the complete assembly of this fusion site.

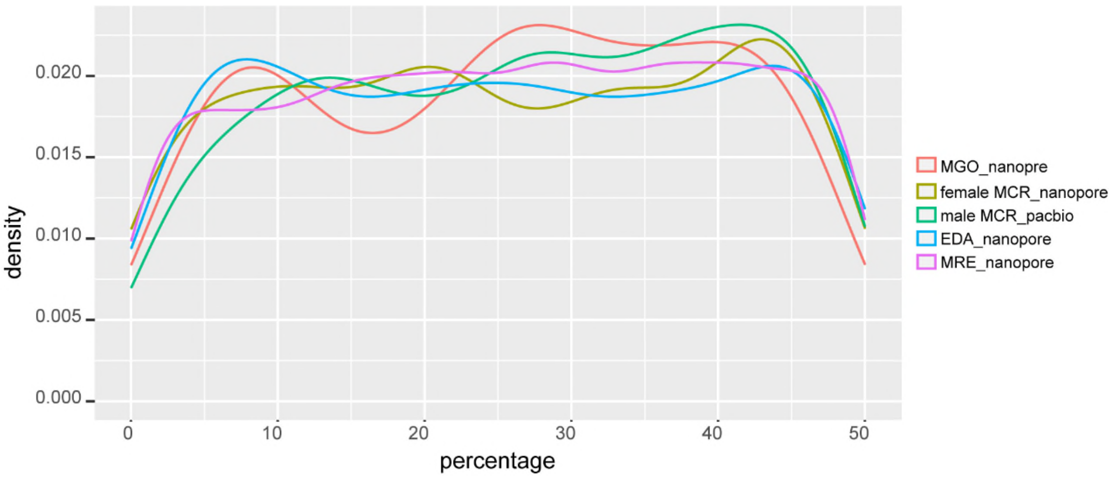

Supplementary Figure 20. Density of reads with different distance between short telomeric repeats from the reads' end. The abscissa shows the percentage of distance between the short telomeric repeat and its nearest read end to the total length of the read. MGO, *M. gongshanensis*; MCR, *M. crinifrons*; EDA, *E. davidianus*; MRE, *M. reevesi*.

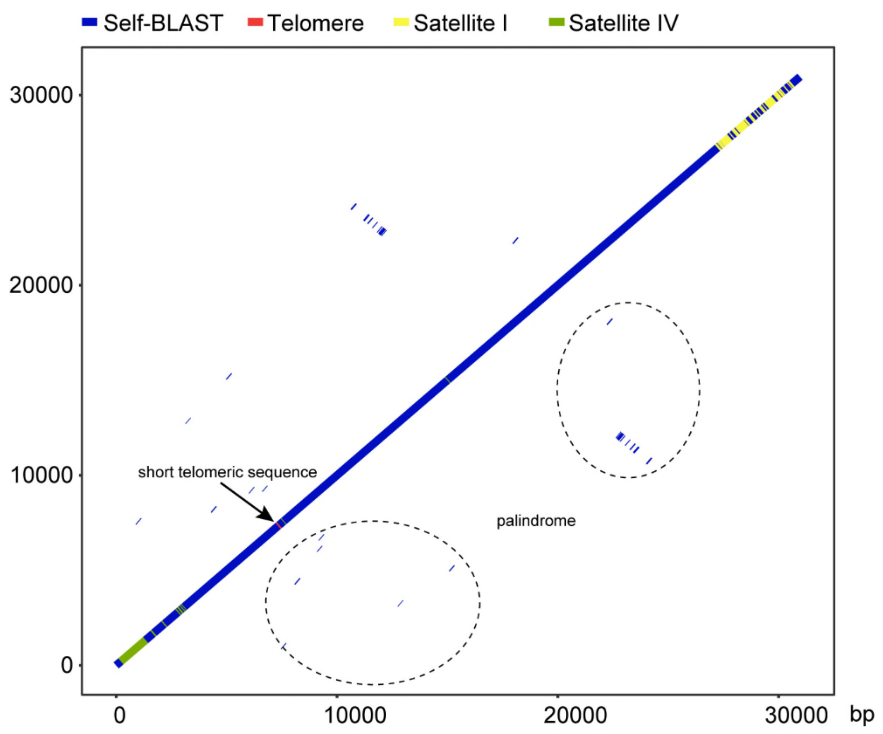

Supplementary Figure 21. An example of reads which can be simultaneously aligned by satellite I, IV and telomeric repeats. One nanopore read, with the id “dd3a0c38-516b-4d43-9f42-1a7db1bf3475”, from the female *M. crinifrons* was aligned with satellite sequences and telomeric repeats, together with its self-alignment, using BLAST. The dark blue short lines distributed on both sides of the diagonal lines are palindrome and are encircled by dotted curve. There is a very short telomeric sequence region on the read, which is marked out by an arrow.

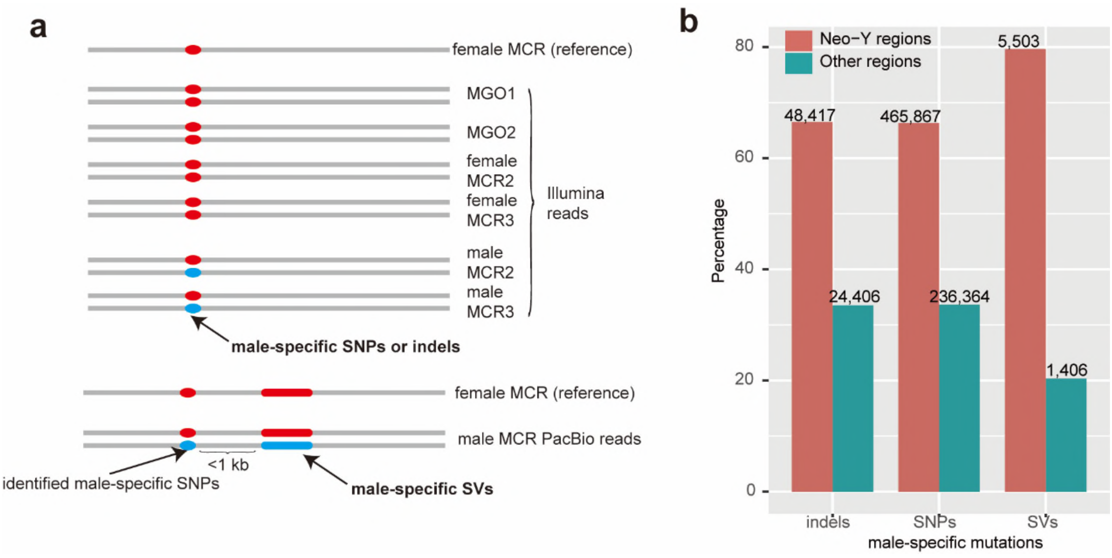

Supplementary Figure 22. Candidate male-specific mutations. a Schematic diagram of identifying candidate male-specific mutations. Gray horizontal lines represent homologous chromosomes, on which red circles or bars mean alleles the same to reference and blue ones mean alterative alleles. On the right side of the gray lines are sample ids. MCR, *M. crinifrons*; MGO, *M. gongshanensis*; SNPs, single nucleotide polymorphisms; indels, insertions and deletions; SVs, structural variations. **b** Proportion and number of candidate male-specific mutations in different genomic regions. “Neo-Y regions” refer to the inverted regions on neo-Y chromosome. “Other regions” refer to the rest genomic regions.

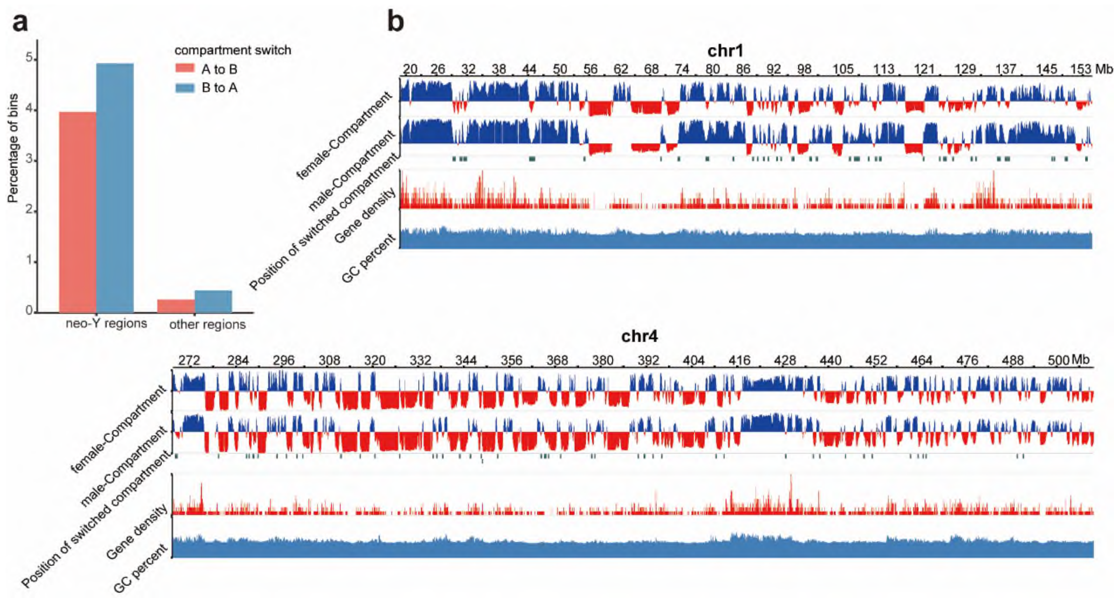

Supplementary Figure 23. Compartment A/B in neo-Y regions. a Proportion of bins with switched compartment type between female and male *M. crinifrons* genome. "A to B" indicate compartment switch from A in female *M. crinifrons* to compartment B in male. "B to A" indicates opposite compartment switch. **b** Compartment A/B of female and male *M. crinifrons*, and other genomic features in neo-Y regions.

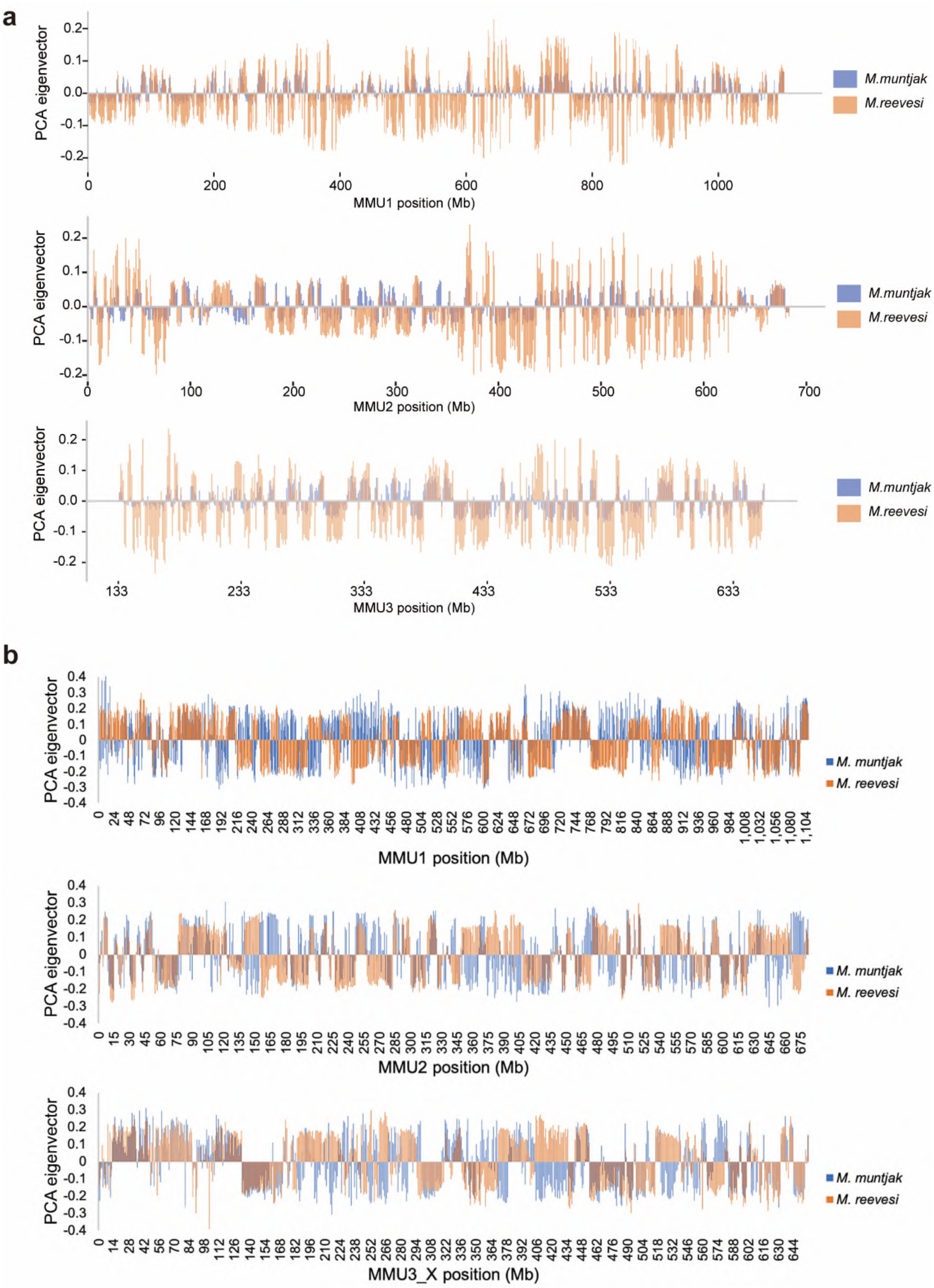

Supplementary Figure 24. Comparison of compartment A/B results from different methods. a Compartment A/B of *M. muntjak vaginalis* and *M. reevesi* identified by method we used in this study. The Hi-C data and genomes of *M. muntjak vaginalis* and *M. reevesi* are all from Mudd et al. [32]. The Hi-C reads of *M. muntjak*

vaginalis and *M. reevesi* against their own reference genomes to identify their compartment A/B separately. The compartment results of *M. reevesi* was then assigned to the genome of *M. muntjak vaginalis* according their bins' homologous mapping relationship. Orange bars are compartment of *M. reevesi* and blue ones are *M. muntjak vaginalis*. Regions where orange and blue do not overlap can be considered as compartment switch between *M. reevesi* and *M. muntjak vaginalis*. Due to the low quality assembly of *M. reevesi*'s X chromosome, we just obtained three homologous bins between X chromosome of *M. reevesi* and X regions of *M. muntjak vaginalis*. The compartment of *M. reevesi* and *M. muntjak vaginalis* in X region could not be displayed comparatively. MMU, *M. muntjak vaginalis*; MRE, *M. reevesi*. **b** The graph showing compartment comparison between *M. reevesi* and *M. muntjak vaginalis* from the supplementary material of Mudd et al's study. In their study, the compartment of *M. reevesi* is identified by aligning *M. reevesi*'s Hi-C reads to *M. muntjak vaginalis* genome.

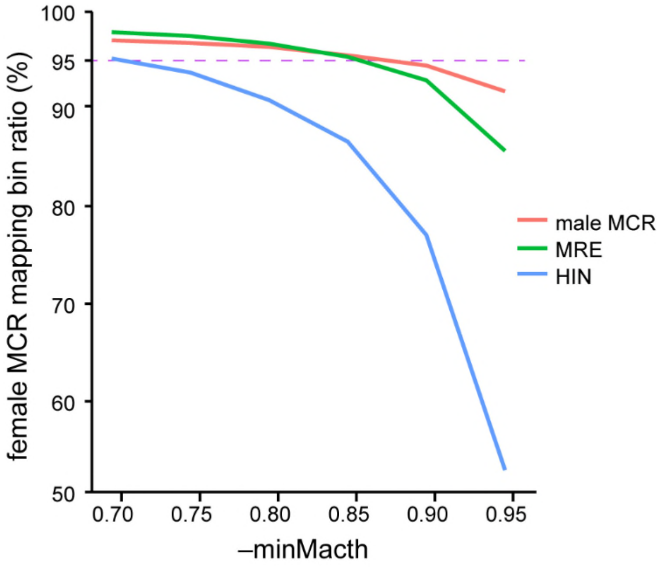

Supplementary Figure 25. Ratios of female *M. crinifrons* bins mapped with homologous bins in other three genomes. The bins are at 40 kb resolution. The horizontal ordinate is the parameter of liftover. MCR, *M. crinifrons*; MRE, *M. reevesi*; HIN, *H. inermis*.

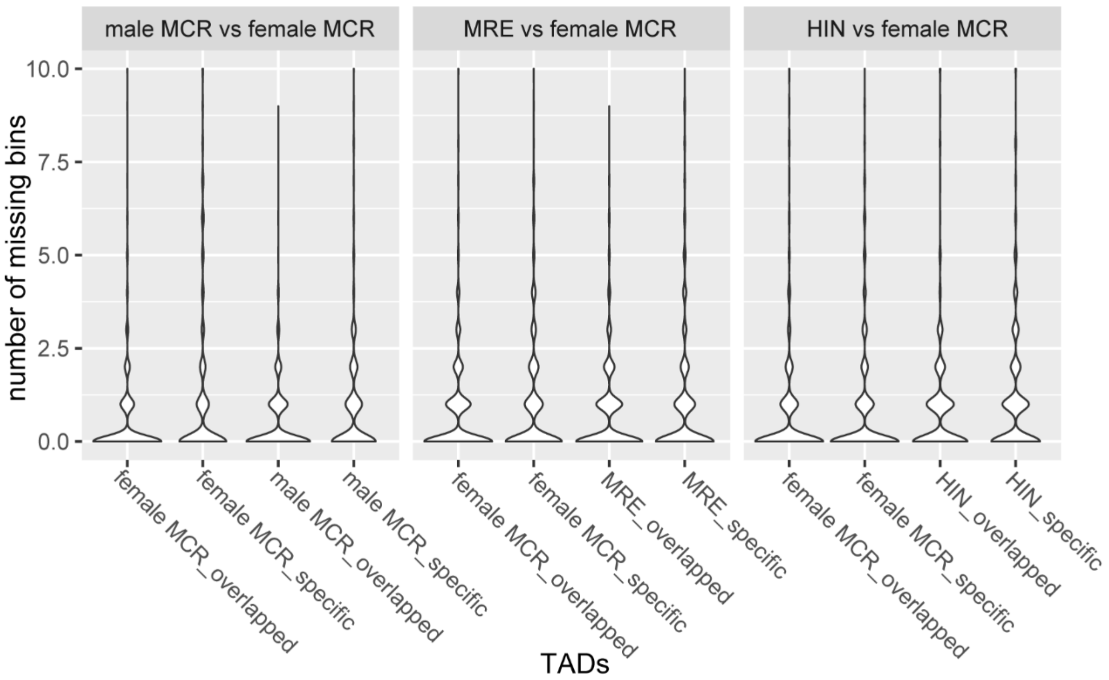

Supplementary Figure 26. Number of missing bins in different type of TADs. "female MCR_overlapped" means female *M. crinifrons* TADs that are homologous with TADs in male *M. crinifrons*, *M. reevesi* (Chinese muntjac) or *H. inermis* (Chinses water deer). "female MCR_specific" means female *M. crinifrons* TADs that are not homologous with TADs in male *M. crinifrons*, *M. reevesi* or *H. inermis*. Similarly, "male MCR_overlapped", "MRE_overlapped" and "HIN_overlapped" respectively represent TADs of male *M. crinifrons*, *M. reevesi*, and *H. inermis* that are homologous with female *M. crinifrons*. "male MCR_specific", "MRE_specific" and "HIN_specific" represent male *M. crinifrons*, *M. reevesi*, and *H. inermis* TADs that are not homologous with female *M. crinifrons*.

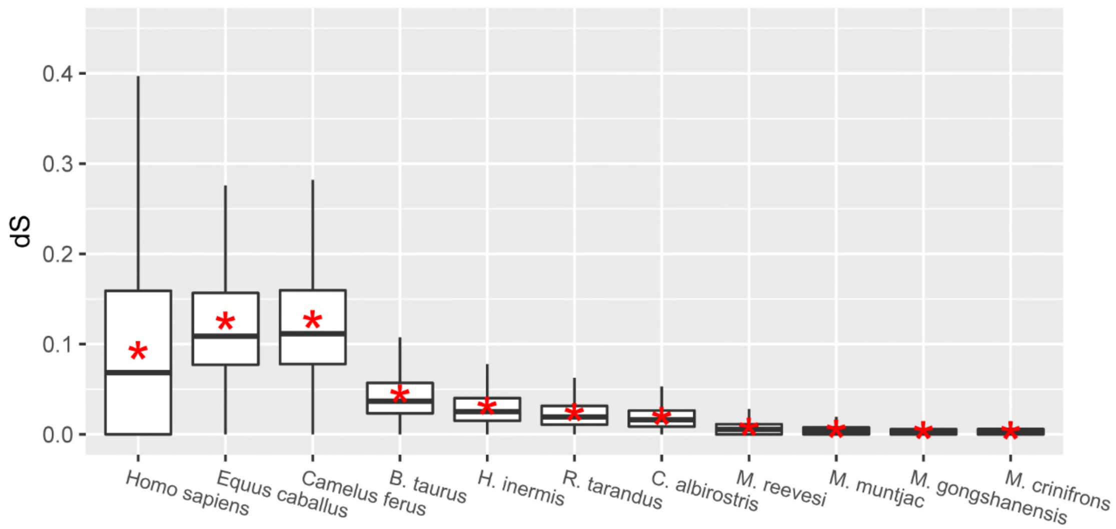

Supplementary Figure 27. Synonymous substitution rate (*dS*) of genes. The *dS* values of 16,627 homologous genes were calculated using the free-ratio model of the Codeml module in PAML software package (version 4.8) [33]. The lower and upper hinges correspond to the first and third quartiles. The horizontal bold line inside the box is the median. The upper whisker extends from the hinge to the largest value no further than 1.5 * IQR from the hinge (where IQR is distance between the first and third quartiles). The lower whisker extends from the hinge to the smallest value at most 1.5 * IQR of the hinge. Outlier are not displayed. The red stars represent the average value of *dS*.

Supplementary Tables

Supplementary Table 1. Information of samples.

Species information, collection locations and sample types are listed here. All the samples are collected in China. The number in sample id represent different individuals.

Species	Sample id	Sample gender	Collection locations	Sample type
H. inermis	HIN1	Male	Jiangsu province	blood
M. reevesi	MER1	Male	Hefei Wild-life Zoo	blood
M. gongshanensis	MGO1	Female	Kunming Cell Bank	frozen tissue
	MGO2	Unknown	Kunming Cell Bank	frozen tissue
M. crinifrons	female MCR1	Female	Hefei Wild-life Zoo	blood
	female MCR2	Female	Hefei Wild-life Zoo	blood
	female MCR3	Female	Kunming Cell Bank	frozen tissue
	female MCR4	Female	Kunming Cell Bank	cell line
	male MCR1	Male	Hefei Wild-life Zoo	blood
	male MCR2	Male	Hefei Wild-life Zoo	blood
	male MCR3	Male	Kunming Cell Bank	frozen tissue
	male MCR4	Male	Kunming Cell Bank	cell line
E. davidianus	EDA1	Male	Beijing Milu Ecological Research Center	blood

Supplementary Table 2. Summary of Nanopore sequencing results.

Reads number, reads N50 length, amounts and coverage of clean data of samples sequenced by Nanopore technology.

**Species**	**Sample id**	**Reads number**	**Reads N50 (kb)**	**Clean data (Gb)**	**Coverage (×)**
M. crinifrons	Female MCR 1	12,165,895	34.9	305.2	122
M. reevesi	MER1	6,841,035	38.1	190.4	76
M. gongshanensis	MGO1	10,322,205	26.2	186.5	75
E. davidianus	EDA1	2,576,866	33.8	62.1	25

Supplementary Table 3. **Summary of next-generation sequencing (NGS) results.** Amounts and coverage of clean data for samples sequenced by next-generation sequencing (Illumina) technology.

**Species**	**Sample id**	**Clean data (Gb)**	**Coverage (X)**
M. crinifrons	Female MCR1	152.0	62
	Female MCR2	154.7	62
	Female MCR3	110.3	44
	Male MCR2	164.6	62
	Male MCR3	106.9	40
M. reevesi	MRE1	127.0	52
M. gongshanensis	MGO1	122.0	49
	MGO2	131.3	53

Supplementary Table 4. Summary of Hi-C sequencing results.

Amounts and coverage of clean data of Hi-C sequencing. Partial (52~72 ×) Hi-C data were used for chromosome assembly.

Species	Sample id	Total data			Data for chromosome assembly	
		Base (Gb)	Coverage (×)	Reads pair	Base (Gb)	Coverage (×)
M. crinifrons	Female MCR1	663.0	266	2,246,086,718	174.01	71
	Male MCR1	797.0	300	2,700,196,562	127.40	52
M. reevesi	MRE1	818.6	328	2,778,092,796	137.76	56
H. inermis	HIN1	669.5	264	3,353,304,975	179.73	72

Supplementary Table 5. **Genome assembly statistics.**

The draft genomes of female *M. crinifrons* (MCR), *M. reevesi* (MRE) and *M. gongshanensis* (MGO) were assembled using newly obtained nanopore reads in this study and that of male *M. crinifrons* were reassembled using the downloaded PacBio reads (PRJNA438286) from [6]. The draft genome of *H. inermis* (HIN) assembled using 10X genomics data is from the study by Wang et al. [34]. "Mounting ratio" means the percentage of contig sequence anchored on chromosomes. Completeness of chromosome or contig assembly are assessed by BUSCO based on the mammalia_odb9 set [26].

**Genomes**	**Female MCR**	**Male MCR**	**MGO**	**MRE**	**HIN**
**Contig N50 (Mb)**	37.86	3.79	24.47	33.32	-
**Num. of contigs**	265	20,390	314	299	-
**Genome size (Gb)**	2.489	2.658	2.475	2.493	2.530
**Scaffold N50 (Mb)**	646.57	535.59	-	113.32	74.97
**Num. of chr.**	4	5	-	23	35
**Mounting ratio (%)**	98.82	91.49	-	98.62	97.57
**Busco (%)**	95.2	94.9	-	95.2	94.9

Supplementary Table 6. Mutation rate and generation time. The mutation rate was calculated using r8s based on the phylogenetic tree with calibrated divergence time. Generation time of the species used in PSMC analysis are listed here. The generation time of *H. inermis* and *C. albirostris* are referenced from Chen et al. [6] and that of the four muntjac species are referenced from Di Marco et al. [35].

Species	Mutation rate	Generation time
B. taurus	1.7406e-09	-
R. tarandus	2.5032e-09	-
H. inermis	3.2651e-09	5
E. davidianus	2.2076e-09	-
C. albirostris	2.0384e-09	5
M. reevesi	2.3796e-09	2.5
M. gongshanensis	2.4537e-09	2.5
M. crinifrons	2.4796e-09	2.5
M. muntjak vaginalis	2.4365e-09	2.5

Supplementary Table 7. Fusion events identified in female *M. crinifrons* genome.

The fusion events in female *M. crinifrons* genome is inferred from the chromosome alignment of female *M. crinifrons* with *B. taurus*, *H. inermis* and *M. reevesi*. Each fusion event on each chromosome of female *M. crinifrons* are marked by the ancestral chromosome represented by chromosome or chromosome segments of *B. taurus*, *H. inermis* and *M. reevesi* which directly fused in female *M. crinifrons*. The “Left” and “Right” respectively label chromosomes directly involved in each fusion event. The “Type” column indicates the type of fusion event, including tandem fusion (T) and Robertsonian fusion (R). The “Class” column indicates that the sharing of fusion events by various muntjac species, which is deduced from the previous cytological results [36-38] and our chromosome synteny results. C_N indicates that fusion events which shared by N muntjac species. Shared by fewer species, the younger the fusion event.

**Chr. of female MCR**	**BTA**		**HIN**		**MRE**		**Type**	**Class**
	**Left**	**Right**	**Left**	**Right**	**Left**	**Right**		
1	19	10	18	5	17	8	T	C_1
1	10	1	5	27	8	18	R	C_1
1	1	29	27	29	18	5a	T	C_3
1	29	8	29	17	5a	19	T	C_4
1	8	12	17	8	19	9	T	C_2
1	12	6	8	19	9	16	T	C_4
1	6	6	19	30	16	21	T	C_4
1	6	4	30	2	21	6	T	C_3
1	4	16	2	12	6	5b	T	C_4
2	18	25	20	33	2d	2c	T	C_5
2	25	26	33	7	2c	2b	T	C_5
2	28	9	7	22	2b	14	T	C_4
2	9	20	22	15	14	13	T	C_4
2	20	21	15	16	13	15	T	C_4
2	21	27	16	32	15	11b	T	C_4
2	27	8	32	31	11b	11a	T	C_5
2	8	15	31	11	11a	10	T	C_3
2	15	1	11	6	10	7	R	C_1
3	13	17	9	14	2a	20	T	C_3
3	17	22	14	21	20	4b	T	C_4

3	22	5	21	26	4b	4c	T	C_5
3	5	3	26	1	4c	1b	T	C_4
3	3	7	1	3	1b	1c	T	C_5
4	11	2	4	25	3d	3c	T	C_5
4	2	14	25	10	3c	12	T	C_4
4	14	23	10	28	12	22	T	C_4
4	23	24	28	23	22	4a	T	C_4
4	24	2	23	13	4a	3a	T	C_3
4	2	5	13	24	3a	1a	T	C_3
4	5	9	24	34	1a	3b	T	C_1
4	9	X	34	X	3b	X	R	C_1

Supplementary Table 8. The statistics of genome alignment results. Total base means the smallest total chromosome length between genome pairs. Alignment ratio is the percentage of alignment length in total base. Sequence identity is the percentage of exact matched base in alignment length. Substitution rate is the number of substituted bases divided by two folds of divergence time.

	female *M. crinifrons*			*C. hircus*	
	male *M. crinifrons*	*M. reevesi*	*H. inermis*	*B. taurus*	*O. aries*
Total base	2,432,301,072	2,459,394,662	2,468,664,404	2,582,134,882	2,582,134,882
Mapped base	2,392,894,791	2,391,481,492	2,279,835,188	2,111,717,083	2,423,998,847
Mapped ratio	98.4%	97.2%	92.3%	81.78%	93.88%
Identical base	2,368,023,317	2,326,978,218	2,112,335,753	1,479,479,959	2,257,529,284
Sequence Identity	98.9%	97.3%	92.7%	70.06%	93.13%
Substitution rate	-	4.4E-09	3.2E-09	10.6E-09	5.7E-09
Divergence time (Mya)	-	3.05	11.33	14.1	6.0

Supplementary Table 9. Positions, length and gaps of 31 fusion sites in female *M. crinifrons*. The regions of fusion sites were inferred from the chromosome synteny between female *M. crinifrons* and *B. taurus* or *H. inermis*. In order to correspond fusion sites to fusion events, we also marked the ancestral chromosome fragments on both sides of fusion sites using the number of chromosome fragments of *M. reevesi*. C_1 to C_5 indicate fusion sites shared by one to five muntjac species, which can also reflect the time stage of fusion site formation. By checking the appearance of Ns and coverage of nanopore reads in fusion sites and their upstream and downstream 200 kb region, we inferred the number of assembly gaps remained in each fusion site.

**Chr**	**Start**	**End**	**Length**	**Left Segment.**	**Right Segment.**	**Class**	**Num. of gaps**
1	60,249,089	60,682,528	433,440	17	8	C_5	1
1	154,881,321	155,184,370	303,050	8	18	C_5	1
1	206,630,177	207,119,107	488,931	18	5a	C_3	2
1	253,998,847	254,650,874	652,028	5a	19	C_2	1
1	315,391,968	315,628,621	236,654	19	9	C_4	2
1	397,714,843	397,805,646	90,804	9	16	C_2	1
1	459,052,872	459,170,307	117,436	16	21	C_2	1
1	506,410,362	506,496,327	85,966	21	6	C_3	1
1	618,388,290	618,550,821	162,532	6	5b	C_2	3
2	61,903,828	61,910,032	6,205	2d	2c	C_1	0
2	102,662,795	102,752,384	89,590	2c	2b	C_1	1
2	193,951,504	194,015,859	64,356	2b	14	C_2	1
2	252,799,496	252,935,771	136,276	14	13	C_2	1
2	320,075,527	320,279,220	203,694	13	15	C_2	1
2	385,662,221	385,787,990	125,770	15	11b	C_2	1
2	427,017,647	427,241,129	223,483	11b	11a	C_1	2
2	470,398,810	470,568,565	169,756	11a	10	C_3	1

2	547,839,849	548,127,395	287,547	10	7	C_5	1
3	79,413,509	79,487,659	74,151	2a	20	C_3	1
3	148,345,652	148,346,381	730	20	4b	C_2	1
3	206,306,444	206,386,443	80,000	4b	4c	C_1	1
3	259,039,164	259,053,513	14,350	4c	1b	C_2	1
3	373,017,011	373,149,282	132,272	1b	1c	C_1	1
4	101,146,058	101,649,364	503,307	3d	3c	C_1	1
4	155,168,668	155,313,264	144,597	3c	12	C_2	1
4	232,690,291	232,843,171	152,881	12	22	C_2	3
4	281,877,412	282,036,727	159,316	22	4a	C_2	1
4	339,822,999	339,997,157	174,159	4a	3a	C_3	1
4	414,079,771	414,217,410	137,640	3a	1a	C_3	1
4	471,938,315	472,086,989	148,675	1a	3b	C_5	2
4	509,909,216	510,430,186	520,971	3b	X	C_5	1

Supplementary Table 10. Nanopore reads used to investigate molecular cause of chromosome fusions. Partial nanopore reads of female *M. crinifrons* (female MCR), *M. reevesi* (MRE), *M. gongshanensis* (MGO) and *E. davidianus* (EDA) were extracted. The number of reads and their total base are listed here. The content of satellite and telomeric sequence in these reads were obtained by aligning three types of Cervidae-specific satellite sequence and telomere of vertebrates to these reads. satI, satellite I; satII, satellite II; satIV, satellite IV.

**Species**		**Female MCR**	**MGO**	**MRE**	**EDA**
**Total reads**		1,669,306	2,000,000	1,696,620	2,576,866
**Total bases**		44,051,645,020	40,591,609,554	46,742,883,242	62,037,303,795
**SatI**	**Bases**	351,913,255	417,925,797	198,999,611	2,907,788,968
	**Ratio (%)**	0.7981	1.0267	0.4207	4.6871
**SatII**	**Bases**	371,947,908	35,070,298	30,027,897	140,736,951
	**Ratio (%)**	0.8347	0.0834	0.0627	0.2268
**SatIV**	**Bases**	1,361,361	870,155	27,123,048	3,149,655
	**Ratio (%)**	0.0027	0.0016	0.0552	0.005
**Telomere**	**Bases**	974,183	769,197	3,948,520	6,464,670
	**ratio (%)**	0.0022	0.0019	0.0084	0.0104

Supplementary Table 11. Number of reads with palindrome sequence at 4 kb upstream and downstream of the truncated short telomeric sequence. In some of the three types of reads containing satellite and short telomeric sequence, we searched for palindrome sequences in the region of 4 kb upstream and downstream of the short telomeric sequence. The total number of the three types of reads, the number and ratio of reads with palindrome sequences were listed here. satI, satellite I sequence; satIV, satellite IV sequence; telomere, telomeric sequence.

**Species**	**Number of reads with palindrome**			**Total reads with palindrome**	**Total reads**	**Ratio (%)**
	**satI-telomere (>=500)**	**satIV-telomere**	**satI-satIV-telomere**			
Female MCR	38	72	450	560	606	92.41
MGO	19	62	113	194	218	88.99
MRE	187	992	2888	4067	4582	88.76
EDA	14	160	277	451	452	99.78

Supplementary Table 12. Number of nanopore reads with different repeat patterns. The nanopore reads aligned with satellite sequences or telomeric sequences were divided into different patterns based on whether they contain a certain satellite sequence or telomeric sequence. For the convenience of recording, we used a four-bit binary number to represent different patterns. From left to right, each bit represents satellite I, satellite II, satellite IV and telomeric sequence, respectively. When a read contains a certain type of satellite sequence or telomeric sequence, the corresponding position is set to 1, otherwise it is 0. For example, pattern 1011 indicates that this read synchronously contains satellite I, satellite IV and telomeric sequence. Then we counted the number of nanopore reads for each pattern. The proportions (0.01%) of reads with different patterns in the total investigated reads are also listed here. MGO, *M. gongshanensis*; MRC, *M. crinifrons*; MRE, *M. reevesi*; EDA, *E. davidianus*.

**pattern**	**Female MCR**		**MGO**		**MRE**		**EDV**	
	**number**	**ratio (0.01%)**	**number**	**ratio (0.01%)**	**number**	**ratio (0.01%)**	**number**	**ratio (0.01%)**
0001	955	5.72	928	4.64	1093	6.44	2610	10.13
0010	1487	8.91	1349	6.75	8438	49.73	3450	13.39
0100	133401	799.14	23143	115.72	14615	86.14	49555	192.31
0110	1164	6.97	76	0.38	877	5.17	2742	10.64
1000	27086	162.26	46629	233.15	40300	237.53	230484	894.44
1010	1884	11.29	741	3.71	19039	112.22	1304	5.06
1100	701	4.20	2991	14.96	22	0.13	385	1.49
1110	12	0.07	30	0.15	13	0.08	16	0.06
0011	75	0.45	67	0.34	1096	6.46	160	0.62
0101	27	0.16	16	0.08	181	1.07	464	1.80
0111	0	0.00	2	0.01	4	0.02	3	0.01
1001	162	0.97	120	0.60	294	1.73	16	0.06
1011	464	2.78	121	0.61	3218	18.97	277	1.07
1101	0	0.00	1	0.01	0	0.00	472	1.83
1111	7	0.04	5	0.03	2	0.01	21	0.08

Supplementary Table 13. The statistics of genomic rearrangements. The genomes of *H. inermis* (HIN), *E. davidianus* (EDA) and four muntjac deer were firstly aligned to the genome of cattle, and then the genomic rearrangements (inter- and intra-chromosome translocations, and inversions) happened in these query genomes were inferred. MRE, *M. reevesi*; MCR, *M. crinifrons*; MGO; *M. gongshanensis*.

**Species**	**Inter-chr**	**Intra-chr**	**Inversion**	**Total rearrangement**	**Total aligned length**	**Rearrangement per Mb**
MGO	727	225	10	962	2471698130	3.89
female MCR	518	236	18	772	2460013611	3.14
male MCR	522	200	23	745	2432301072	3.06
EDV	720	404	15	1139	2500365215	4.56
MRE	533	222	9	764	2459394662	3.11
HIN	663	237	54	954	2468664404	3.86

Supplementary Table 14. Genomic position of the neo-sex regions. According to chromosome collinearity among female *M. crinifrons*, male *M. crinifrons, M. reevesi*, *H. inermis* and *B. taurus* and the previous studies about neo-Y chromosome in BM [36], we inferred the detailed genomic position of the neo-Y region in male *M. crinifrons* and its homologous regions (neo-X regions) in female *M. crinifrons*. The rest region except for the neo-Y region in the 1p+4 chromosome is regarded as "Other regions". The ancestral chromosome segments were labeled according to the chromosomal regions of *M. reevesi*. The coordinates of the start and end positions and region length are in Mb.

**Regions**	**labels**	**male *M. crinifrons***				**female *M. crinifrons***			
		**Chr**	**Start**	**End**	**Length**	**Chr**	**Start**	**End**	**Length**
Other	3d+3c+12+22b	1p+4	0.00	267.12	267.12	X+4	0.00	267.49	267.49
Neo-sex	17a	1p+4	267.12	308.50	41.38	1	18.98	60.57	41.60
Neo-sex	3b	1p+4	308.50	346.64	38.14	X+4	472.09	509.93	37.83
Neo-sex	8	1p+4	346.64	438.09	91.45	1	60.67	154.91	94.24
Neo-sex	22a+4a+3a+1a	1p+4	438.09	642.30	204.21	X+4	267.50	472.07	204.57
Other	17b	1p+4	642.30	661.25	18.95	1	0.00	18.96	18.96

Supplementary Table 15. Distribution of candidate male-specific mutations annotated as “MODERATE”. We counted the number of “MODERATE” mutations in different genomic regions, including neo-Y region and the rest other regions. The total length of coding sequence (cds) of all genes in each region was also calculated. Within each 1 kb, the number of "MODERATE" mutations is shown here.

**Region**	**Mutation number**	**Cds total length**	**Count/kb**
Neo-Y regions	1589	5,707,376	0.278
Other regions	687	28,476,382	0.024

Supplementary Table 16. Comparison of topologically associating domain (TAD) between female and male *M. crinifrons*. The number of total TADs, altered TADs and conserved TADs between female and male *M. crinifrons* in the neo-Y region and all other genomic regions are listed here.

**Region**	**Num. of altered TADs**	**Num. of conservative TADs**	**Total TADs**
Neo-Y region	103	311	414
Other regions	571	1467	2038
Total	674	1778	2452

Supplementary Table 17. Statistic of the Hi-C reads alignment results. The Hi-C reads and genomes of *M. muntjak vaginalis* and *M. reevesi* sequenced and assembled by Mudd et al. [32] were downloaded from NCBI (PRJNA542135 and PRJNA542137). The Hi-C reads of *M. muntjak vaginalis* were aligned to *M. muntjak vaginalis* itself genome, while that of *M. reevesi* were aligned to the *M. muntjak vaginalis* genome like Mudd et al. as well aligned to *M. reevesi* genome using the HiC-Pro pipeline [39].

	**MMU to MMU**		**MRE to MMU**		**MRE to MRE**	
**Alignments results**	**Reads number**	**Reads ratio**	**Reads number**	**Reads ratio**	**Reads number**	**Reads ratio**
Total pairs	260874784	100	265001043	100	265001043	100
Unmapped pairs	20348755	7.774	37892491	14.227	27222433	10.197
Low qual pairs	36456945	13.854	82360963	31.099	40440626	15.143
Unique paired alignments	177526233	68.281	70227680	26.636	156081943	59.373
Multiple pairs alignments	0	0	0	0	0	0
Pairs with singleton	26542851	10.09	74519909	28.038	41256041	15.288
Low qual singleton	0	0	0	0	0	0
Unique singleton alignments	0	0	0	0	0	0
Multiple singleton alignments	0	0	0	0	0	0
Reported pairs	177526233	68.281	70227680	26.636	156081943	59.373

Supplementary Table 18. Number of PSGs and REGs under different filtering criterion. The *p*-values are from one-sided chi-square test (*p*-value <0.05) and the fdr value are adjusted *p*-value using “fdr” method (FDR < 0.05). “MCR_MGO_MMU” indicate that the PSGs and the REGs are identified at the common ancestor of *M. crinifrons* (MCR), *M. gongshanensis* (MGO) and *M. muntjak vaginalis* (MMU).

	p-value <0.05		FDR <0.05	
Lineages	PSGs	REGs	PSGs	REGs
MCR_MGO_MMU	32	210	1	9
MCR	72	509	6	17
MGO	70	582	8	21
MMU	131	611	18	38

References

1. Liu, H., Wu, S., Li, A. & Ruan, J. SMARTdenovo: A de novo Assembler Using Long Noisy Reads. *Preprints 2020* **2020090207**.

2. Hu, J., Fan, J., Sun, Z. & Liu, S. NextPolish: a fast and efficient genome polishing tool for long-read assembly. *Bioinformatics* **36**, 2253-2255, (2020).

3. Vaser, R., Sovic, I., Nagarajan, N. & Sikic, M. Fast and accurate de novo genome assembly from long uncorrected reads. *Genome Res* **27**, 737-746, (2017).

4. Walker, B. J. *et al.* Pilon: an integrated tool for comprehensive microbial variant detection and genome assembly improvement. *PLoS One* **9**, e112963, (2014).

5. Ruan, J. & Li, H. Fast and accurate long-read assembly with wtdbg2. *Nature Methods*, (2019).

6. Chen, L. *et al.* Large-scale ruminant genome sequencing provides insights into their evolution and distinct traits. *Science* **364**, (2019).

7. Durand, N. C. *et al.* Juicer Provides a One-Click System for Analyzing Loop-Resolution Hi-C Experiments. *Cell Syst* **3**, 95-98, (2016).

8. Dudchenko, O. *et al.* De novo assembly of the Aedes aegypti genome using Hi-C yields chromosome-length scaffolds. *Science* **356**, 92-95, (2017).

9. Durand, N. C. *et al.* Juicebox Provides a Visualization System for Hi-C Contact Maps with Unlimited Zoom. *Cell Syst* **3**, 99-101, (2016).

10. Kielbasa, S. M., Wan, R., Sato, K., Horton, P. & Frith, M. C. Adaptive seeds tame genomic sequence comparison. *Genome Res* **21**, 487-493, (2011).

11. Krzywinski, M. *et al.* Circos: an information aesthetic for comparative genomics. *Genome Res* **19**, 1639-1645, (2009).

12. Benson, G. Tandem repeats finder: a program to analyze DNA sequences. *Nucleic Acids Res* **27**, 573-580, (1999).

13. Xu, Z. & Wang, H. LTR_FINDER: an efficient tool for the prediction of full-length LTR retrotransposons. *Nucleic Acids Res* **35**, W265-268, (2007).

14. Smit, A., Hubley, R. & Green, P. RepeatMasker Open-4.0. (2013-2015).

15. Bao, W., Kojima, K. K. & Kohany, O. Repbase Update, a database of repetitive elements in eukaryotic genomes. *Mob DNA* **6**, 11, (2015).

16. Korf, I. Gene finding in novel genomes. *BMC Bioinformatics* **5**, 59, (2004).

17. Burge, C. & Karlin, S. Prediction of complete gene structures in human genomic DNA. *J Mol Biol* **268**, 78-94, (1997).

18. Majoros, W. H., Pertea, M. & Salzberg, S. L. TigrScan and GlimmerHMM: two open source ab initio eukaryotic gene-finders. *Bioinformatics* **20**, 2878-2879, (2004).

19. Stanke, M. *et al.* AUGUSTUS: ab initio prediction of alternative transcripts. *Nucleic Acids Res* **34**, W435-439, (2006).

20. Pennisi, E. The Human Genome. *Science* **291**, 1177-1180, (2001).

21. Zimin, A. V. *et al.* A whole-genome assembly of the domestic cow, Bos taurus. *Genome biology* **10**, R42, (2009).

22. Altschul, S. F. *et al.* Gapped BLAST and PSI-BLAST: a new generation of protein database search programs. *Nucleic Acids Res* **25**, 3389-3402, (1997).

23. She, R., Chu, J. S., Wang, K., Pei, J. & Chen, N. GenBlastA: enabling BLAST to identify homologous gene sequences. *Genome Res* **19**, 143-149, (2009).

24. Birney, E., Clamp, M. & Durbin, R. GeneWise and Genomewise. *Genome Res* **14**, 988-995, (2004).

25. Haas, B. J. *et al.* Automated eukaryotic gene structure annotation using EVidenceModeler and the program to assemble spliced alignments. *Genome biology* **9**, (2008).

26. Simao, F. A., Waterhouse, R. M., Ioannidis, P., Kriventseva, E. V. & Zdobnov, E. M. BUSCO: assessing genome assembly and annotation completeness with single-copy orthologs. *Bioinformatics* **31**, 3210-3212, (2015).

27. Camacho, C. *et al.* BLAST+: architecture and applications. *BMC Bioinformatics* **10**, 421, (2009).

28. Li, H. & Durbin, R. Fast and accurate short read alignment with Burrows-Wheeler transform. *Bioinformatics* **25**, 1754-1760, (2009).

29. Li, H. *et al.* The Sequence Alignment/Map format and SAMtools. *Bioinformatics*

25, 2078-2079, (2009).

30. Thorvaldsdottir, H., Robinson, J. T. & Mesirov, J. P. Integrative Genomics Viewer (IGV): high-performance genomics data visualization and exploration. *Brief Bioinform* **14**, 178-192, (2013).

31. Liu, Y., Nie, W., Huang, L., Wang, J. & Wei-Ting, S. Cloning, characterization, and FISH mapping of four satellite DNAs from black muntjac (M. crinifrons) and Fea's muntjac (M. feae) *Zoological Research* **29**, 225-235, (2008).

32. Mudd, A. B., Bredeson, J. V., Baum, R., Hockemeyer, D. & Rokhsar, D. S. Analysis of muntjac deer genome and chromatin architecture reveals rapid karyotype evolution. *Commun Biol* **3**, 480, (2020).

33. Yang, Z. PAML 4: phylogenetic analysis by maximum likelihood. *Mol Biol Evol* **24**, 1586-1591, (2007).

34. Wang, Y. *et al.* Genetic basis of ruminant headgear and rapid antler regeneration. *Science* **364**, (2019).

35. Di Marco, M. *et al.* Generation length for mammals. *Nature Conservation* **5**, 89-94, (2013).

36. Huang, L. *et al.* High-density comparative BAC mapping in the black muntjac (Muntiacus crinifrons): molecular cytogenetic dissection of the origin of MCR 1p+4 in the X1X2Y1Y2Y3 sex chromosome system. *Genomics* **87**, 608-615, (2006).

37. Chi, J. X. *et al.* Defining the orientation of the tandem fusions that occurred during the evolution of Indian muntjac chromosomes by BAC mapping. *Chromosoma* **114**, 167-172, (2005).

38. Huang, L., Wang, J., Nie, W., Su, W. & Yang, F. Tandem chromosome fusions in karyotypic evolution of Muntiacus: evidence from M. feae and M. gongshanensis. *Chromosome research : an international journal on the molecular, supramolecular and evolutionary aspects of chromosome biology* **14**, 637-647, (2006).

39. Servant, N. *et al.* HiC-Pro: an optimized and flexible pipeline for Hi-C data processing. *Genome biology* **16**, 259, (2015).
