# Supplementary material for: Molecular mechanisms and topological consequences of drastic chromosomal rearrangements of muntjac deer
Source: Nat Commun. 2021 Nov 25;12:6858. doi: 10.1038/s41467-021-27091-0 (PMC8617201; doi:10.1038/s41467-021-27091-0)
Supplement: Supplementary file 3 — Description of Additional Supplementary Files [file 41467_2021_27091_MOESM3_ESM.pdf]

Description of Supplementary Files

File Name: Supplementary Data 1.
Description: Genes at ends of significant interactions crossing fusion sites (SIAFS).

File Name: Supplementary Data 2.
Description: GO and pathway enrichment results of genes at the ends of significant interactions across fusion sites.

File Name: Supplementary Data 3.
Description: The positively selected genes and the rapidly evolving genes in different muntjac lineages.

File Name: Supplementary Data 4.
Description: GO and pathway enrichment results from combined positively selected genes and rapidly evolving genes.

File Name: Supplementary Data 5.
Description: Genes and phenotype related to chromosome stability in Mouse Genome Informatics (MGI) database.

File Name: Supplementary Data 6.
Description: Expression information of genes with disrupted ORFs in neo-Y regions.

File Name: Supplementary Data 7.
Description: Information of genes in the neo-sex regions with switched compartment.

File Name: Supplementary Data 8.
Description: GO and pathway enrichment results of genes in neo-sex regions with switched compartment.
