# Supplementary material for: Molecular mechanisms and topological consequences of drastic chromosomal rearrangements of muntjac deer
Source: Nat Commun. 2021 Nov 25;12:6858. doi: 10.1038/s41467-021-27091-0 (PMC8617201; doi:10.1038/s41467-021-27091-0)
Supplement: Supplementary file 12 — Reporting Summary [file 41467_2021_27091_MOESM12_ESM.pdf]

nature portfolio

Corresponding author(s): Fuwen Wei

Last updated by author(s): Oct 17, 2021

Reporting Summary

Nature Portfolio wishes to improve the reproducibility of the work that we publish. This form provides structure for consistency and transparency in reporting. For further information on Nature Portfolio policies, see our Editorial Policies and the Editorial Policy Checklist.

Statistics

For all statistical analyses, confirm that the following items are present in the figure legend, table legend, main text, or Methods section.

n/a	Confirmed	
[ ]	[x]	The exact sample size (*n*) for each experimental group/condition, given as a discrete number and unit of measurement
[ ]	[x]	A statement on whether measurements were taken from distinct samples or whether the same sample was measured repeatedly
[ ]	[x]	The statistical test(s) used AND whether they are one- or two-sided<br>*Only common tests should be described solely by name; describe more complex techniques in the Methods section.*
[x]	[ ]	A description of all covariates tested
[ ]	[x]	A description of any assumptions or corrections, such as tests of normality and adjustment for multiple comparisons
[x]	[ ]	A full description of the statistical parameters including central tendency (e.g. means) or other basic estimates (e.g. regression coefficient) AND variation (e.g. standard deviation) or associated estimates of uncertainty (e.g. confidence intervals)
[ ]	[x]	For null hypothesis testing, the test statistic (e.g. *F*, *t*, *r*) with confidence intervals, effect sizes, degrees of freedom and *P* value noted<br>*Give P values as exact values whenever suitable.*
[x]	[ ]	For Bayesian analysis, information on the choice of priors and Markov chain Monte Carlo settings
[x]	[ ]	For hierarchical and complex designs, identification of the appropriate level for tests and full reporting of outcomes
[x]	[ ]	Estimates of effect sizes (e.g. Cohen's *d*, Pearson's *r*), indicating how they were calculated

Our web collection on statistics for biologists contains articles on many of the points above.

Software and code

Policy information about availability of computer code

Data collection

The Nanopore, Illumina, Hi-C and RNA-seq data were generated according to their manufacture's instructions.

Data analysis

We listed here the software URLs of all open-source tools used for data analysis.
3D-DNA (version 180922): https://github.com/aidenlab/3d-dna
AUGUSTUS (version 2.5.5): http://augustus.gobics.de/binaries/
bcftools (version 1.3.1): http://samtools.sourceforge.net
bedtools (version 2.25.0): https://github.com/arq5x/bedtools2
BLAST (version 2.2.26): ftp://ftp.ncbi.nlm.nih.gov/blast/executables/blast+/LATEST/
bowrie2 (version 2.2.9): https://github.com/BenLangmead/bowtie2
BUSCO (version 3.0.2): https://busco.ezlab.org/
bwa (version 0.7.12): https://github.com/lh3/bwa
Circos (version 0.69-6): http://circos.ca/software/download/circos/
cworl-dekker (version 1.01): https://github.com/dekkerlab/cworld-dekker
DESeq2 (version 1.20.0): https://bioconductor.org/packages/release/bioc/html/DESeq2.html
EVidenceModeler (version 1.1.1): https://sourceforge.net/projects/evidencemodeler/
fithic (version 1.1.3): https://github.com/ay-lab/fithic
GATK (version 4.1.2.0): https://gatk.broadinstitute.org
GenBlatA (version 22-Sep-2008) http://genome.sfu.ca/genblast/download.html
GeneWise (version 2.2.0): https://www.ebi.ac.uk/Tools/psa/genewise/
TRF (version 4.07b): https://tandem.bu.edu/trf/trf.html
vcftools (version 0.1.13): https://sourceforge.net/projects/vcftools/
wtdbg2 (version 2.5): https://github.com/ruanjue/wtdbg2

GENSCAN (version 1.0): http://gnomic.stanford.edu/GENSCANW.html
GlimmerHMM (version 3.0.4): https://ccb.jhu.edu/software/glimmerhmm/dl/GlimmerHMM-3.0.4.tar.gz
HiC-Pro (version 2.11.4): https://github.com/nservant/HiC-Pro
hisat2 (version 2.1.0): http://daehwankimlab.github.io/hisat2/
igvtools (version 2.3.75): http://software.broadinstitute.org/software/igv/igvtools
juicer (version 1.5.7): https://github.com/aidenlab/juicer
LAST (version 885): http://last.cbrc.jp
liftOver (ucsc tools): http://hgdownload.cse.ucsc.edu/admin/exe/linux.x86_64/
LTR_FINDER (version 1.0.5): http://tlife.fudan.edu.cn/ltr_finder/
MCcan (python version): https://github.com/tanghaibao/jcvi/wiki/MCscan-(Python-version)
metascape (version 3.5): https://metascape.org/gp/index.html#/main/step1
MITObim (version 1.8): https://github.com/chrishah/MITObim
MULTIZ (version 11.2): https://github.com/multiz/multiz
MUSCLE (version 3.8.31): http://www.drive5.com/muscle
Nextpolish (version 1.2.4): https://github.com/Nextomics/NextPolish
PAML (version 4.8): http://web.mit.edu/6.891/www/lab/paml.html#download
Pastis (versoin 0.1.0): https://github.com/hiclib/pastis
picard (version 1.119): https://broadinstitute.github.io/picard/
pilon (version 1.23): https://github.com/broadinstitute/pilon
PSMC (version 0.6.5-r67): http://github.com/lh3/psmc
PyMOL (version 4.5.0): http://www.pymol.org
r8s (version 1.70): http://ginger.ucdavis.edu/r8s
racon (version 1.21): https://github.com/lbcb-sci/racon
RAxML (version 8.2.9): https://github.com/stamatak/standard-RAxML
RepeatMasker (version 4.0.5): http://repeatmasker.org/RepeatMasker/
RepeatModeler (version 1.0.4): http://www.repeatmasker.org/RepeatModeler/
R (version 3.2.5): https://www.r-project.org/
samtools (version 1.3.1): https://github.com/samtools/samtools
smartdenovo (version 1.0): https://github.com/ruanjue/smartdenovo
SNAP (version 2006-07-28): https://sourceforge.net/projects/snap-genefinder/
sniffles (version 1.0.12a): https://github.com/Nextomics/Sniffles
SnpEff (version 4.10): https://pcingola.github.io/SnpEff/
In addition, we also have deposited the in-house code used in this study to the GitHub and can be assessed at https://github.com/YinYuan-001/muntjac_code.

For manuscripts utilizing custom algorithms or software that are central to the research but not yet described in published literature, software must be made available to editors and reviewers. We strongly encourage code deposition in a community repository (e.g. GitHub). See the Nature Portfolio guidelines for submitting code & software for further information.

Data

Policy information about availability of data

All manuscripts must include a data availability statement. This statement should provide the following information, where applicable:

- Accession codes, unique identifiers, or web links for publicly available datasets
- A description of any restrictions on data availability
- For clinical datasets or third party data, please ensure that the statement adheres to our policy

The sequencing data generated in this study have been deposited in the NCBI Sequence Read Archive under accession number PRJNA640966 [https://www.ncbi.nlm.nih.gov/bioproject/640966]. The PacBio data of male *M. crinifrons* used in this study are available in the NCBI Sequence Read Archive under accession number PRJNA438286 [https://www.ncbi.nlm.nih.gov/bioproject/438286].

Field-specific reporting

Please select the one below that is the best fit for your research. If you are not sure, read the appropriate sections before making your selection.

☒ Life sciences ☐ Behavioural & social sciences ☐ Ecological, evolutionary & environmental sciences

For a reference copy of the document with all sections, see nature.com/documents/nr-reporting-summary-flat.pdf

Life sciences study design

All studies must disclose on these points even when the disclosure is negative.

Sample size	In this study, one *H. inermis* (HIN), one *M. reevesi* (MRE), two *M. gongshanensis* (MGO), six *M. crinifrons* (MCR) and one *E. davidianus* (EDA) samples were used to perform genome-wide de novo sequencing and resequencing analysis. These samples were sufficient to generate high quality genomes and perform the comparative genome analysis. In addition, two male and female samples of MCR and two most related species MGO were used to detect the male-specific mutation of M.crinifrons. These samples were not absolutely sufficient to accurately identify the male-specific mutations of *M. crinifrons*, the "candidate male-specific mutation" was used in our manuscript.
Data exclusions	No data were excluded from the analysis.
Replication	Comparative genomics was performed on genomic DNA which is stable in time so that reproducibility was not used in these analyses. *M. crinifrons* is a endangered species, so more transcriptome samples of it are unattainable.
Randomization	Randomization was not applicable because no experimentation was performed in this study.
Blinding	Blinding was not applicable because no experimentation was performed in this study.

Reporting for specific materials, systems and methods

We require information from authors about some types of materials, experimental systems and methods used in many studies. Here, indicate whether each material, system or method listed is relevant to your study. If you are not sure if a list item applies to your research, read the appropriate section before selecting a response.

Materials & experimental systems

n/a	Involved in the study	
☒	☐	Antibodies
☐	☒	Eukaryotic cell lines
☒	☐	Palaeontology and archaeology
☐	☒	Animals and other organisms
☒	☐	Human research participants
☒	☐	Clinical data
☒	☐	Dual use research of concern

Methods

n/a	Involved in the study	
☒	☐	ChIP-seq
☒	☐	Flow cytometry
☒	☐	MRI-based neuroimaging

Eukaryotic cell lines

Policy information about cell lines

Cell line source(s)	Cultured fibroblast cell lines from embryonic lung of female *M. crinifrons* (KCB81002E)<br>Cultured fibroblast cell lines from embryonic skin of male *M. crinifrons* (KCB200004).
Authentication	The cells were provided by Kunming Cell Bank, State Key Laboratory of Genetic Resources and Evolution, Kunming Institute of Zoology, Chinese Academy of Sciences, Kunming, China. The cells were authenticated by amplifying and sequencing the CO1 gene on the mitochondrial genome.
Mycoplasma contamination	The cells tested negative for mycoplasma
Commonly misidentified lines (See ICLAC register)	No commonly misidentified lines used in this study

Animals and other organisms

Policy information about studies involving animals; ARRIVE guidelines recommended for reporting animal research

Laboratory animals	Study did not involve laboratory animals.
Wild animals	The blood samples were come from wild animals or their descendants raised in zoos which were rescued from protected areas. Frozen tissue samples come from wild animals who died in zoos, protected area or in the wild.
Field-collected samples	Study did not collected samples in the field.
Ethics oversight	Our animal experiments were approved by the Committee for Animal Experiments of the Institute of Zoology, Chinese Academy of Science, China (IOZ-IACUC-2021-145).

Note that full information on the approval of the study protocol must also be provided in the manuscript.
